# Operative vs Nonoperative Treatment of Distal Radius Fractures in Adults: A Systematic Review and Meta-analysis

**DOI:** 10.1001/jamanetworkopen.2020.3497

**Published:** 2020-04-23

**Authors:** Yassine Ochen, Jesse Peek, Detlef van der Velde, Frank J. P. Beeres, Mark van Heijl, Rolf H. H. Groenwold, R. Marijn Houwert, Marilyn Heng

**Affiliations:** 1 Department of Orthopedic Surgery, Harvard Medical School Orthopedic Trauma Initiative, Massachusetts General Hospital, Boston; 2 Department of Surgery, University Medical Center Utrecht, Utrecht, the Netherlands; 3 Department of Clinical Epidemiology, Leiden University Medical Center, Leiden, the Netherlands; 4 Department of Surgery, St Antonius Hospital, Nieuwegein, the Netherlands; 5 Department of Surgery, Luzerner Kantonsspital, Luzern, Switzerland; 6 Department of Surgery, Diakonessenhuis Utrecht, Utrecht, the Netherlands

## Abstract

**Importance:**

No consensus has been reached to date regarding the optimal treatment for distal radius fractures. The international rate of operative treatment has been increasing, despite higher costs and limited functional outcome evidence to support this shift.

**Objectives:**

To compare functional, clinical, and radiologic outcomes after operative vs nonoperative treatment of distal radius fractures in adults.

**Data Sources:**

The PubMed/MEDLINE, Embase, CENTRAL (Cochrane Central Register of Controlled Trials), and CINAHL (Cumulative Index to Nursing and Allied Health Literature) databases were searched from inception to June 15, 2019, for studies comparing operative vs nonoperative treatment of distal radius fractures.

**Study Selection:**

Randomized clinical trials (RCTs) and observational studies reporting on the following: acute distal radius fracture with operative treatment (internal or external fixation) vs nonoperative treatment (cast immobilization, splinting, or bracing); patients 18 years or older; and functional outcome. Studies in a language other than English or reporting treatment for refracture were excluded.

**Data Extraction and Synthesis:**

Data extraction was performed independently by 2 reviewers. Effect estimates were pooled using random-effects models and presented as risk ratios (RRs) or mean differences (MDs) with 95% CIs. Data were analyzed in September 2019.

**Main Outcomes and Measures:**

The primary outcome measures included medium-term functional outcome measured with the Disabilities of the Arm, Shoulder and Hand questionnaire (DASH) and the overall complication rate after operative and nonoperative treatment.

**Results:**

A total of 23 unique studies were included, consisting of 8 RCTs and 15 observational studies, that described 2254 unique patients. Among the studies that presented sex data, 1769 patients were women [80.6%]. Overall weighted mean age was 67 [range, 22-90] years). The RCTs included 656 patients (29.1%); observational studies, 1598 patients (70.9%). The overall pooled effect estimates the showed a significant improvement in medium-term (≤1 year) DASH score after operative treatment compared with nonoperative treatment (MD, −5.22 [95% CI, −8.87 to −1.57]; *P* = .005; *I*^2^ = 84%). No difference in complication rate was observed (RR, 1.03 [95% CI, 0.69-1.55]; *P* = .87; *I*^2^ = 62%). A significant improvement in grip strength was noted after operative treatment, measured in kilograms (MD, 2.73 [95% CI, 0.15-5.32]; *P* = .04; *I*^2^ = 79%) and as a percentage of the unaffected side (MD, 8.21 [95% CI, 2.26-14.15]; *P* = .007; *I*^2^ = 76%). No improvement in medium-term DASH score was found in the subgroup of studies that only included patients 60 years or older (MD, −0.98 [95% CI, −3.52 to 1.57]; *P* = .45; *I*^2^ = 34%]), compared with a larger improvement in medium-term DASH score after operative treatment in the other studies that included patients 18 years or older (MD, −7.50 [95% CI, −12.40 to −2.60]; *P* = .003; *I*^2^ = 77%); the difference between these subgroups was statically significant (test for subgroup differences, *P* = .02).

**Conclusions and Relevance:**

This meta-analysis suggests that operative treatment of distal radius fractures improves the medium-term DASH score and grip strength compared with nonoperative treatment in adults, with no difference in overall complication rate. The findings suggest that operative treatment might be more effective and have a greater effect on the health and well-being of younger, nonelderly patients.

## Key Points

**Question:**

What outcomes are associated with operative vs nonoperative treatment of distal radius fractures in adults?

**Findings:**

This meta-analysis of 2254 unique participants in 23 unique studies showed that operative treatment of distal radius fractures improved the medium-term Disabilities of the Arm, Shoulder and Hand questionnaire score and grip strength compared with nonoperative treatment in adults, with no difference in overall complication rate.

**Meaning:**

These findings suggest that operative treatment might be preferred for distal radius fractures.

## Teaser

This meta-analysis compares functional, clinical, and radiologic outcomes after operative vs nonoperative treatment of distal radial fractures in adults.

## Introduction

The fracture of the distal radius is the most common injury in adults, accounting for approximately 17.5% of fractures.^1^ Distal radius fractures have a bimodal age distribution in the population, with a peak incidence seen in patients younger than 18 years and a second peak in patients 50 years or older. Recent studies indicate the worldwide incidence of distal radius fractures is increasing each year owing to the overall potential to live longer with comorbidities such as osteoporosis.^2^ Although the elderly population is at greatest risk, distal radius fractures still have a significant effect on the health and well-being of nonelderly adults. Reports have shown a significant increase of distal radius fractures in patients aged 17 to 64 years.^2^

The management of distal radius fractures consists of operative or nonoperative treatment. However, no consensus has been reached regarding the optimal treatment method. Several meta-analyses have been published on the comparison between operative and nonoperative treatment.^3,4,5^ Recent meta-analyses have focused specifically on patient populations 60 years or older.^4,5^ These meta-analyses found no difference in functional outcome between operative and nonoperative treatment in elderly patients. However, the international rate of operative treatment of distal radius fractures has been increasing, despite higher cost and limited functional outcome evidence to support this shift.^6^

At present, no meta-analysis, to our knowledge, has evaluated functional outcome in patients younger than 60 years by including all patients 18 years or older. Moreover, the high incidence of distal radius fractures and the inconsistencies in treatment practices indicate further investigation is warranted to understand current treatment methods and outcomes.^7^

Randomized clinical trials (RCTs) and observational studies are both increasingly used in orthopedic trauma meta-analyses for the evaluation of treatment effects.^8,9,10,11,12^ Growing evidence shows that meta-analyses of RCTs and observational studies can be of value compared with meta-analyses of RCTs alone. Provided that observational studies are of high quality, the addition of observational studies in meta-analyses increases sample size and might provide a better insight into small treatment effects and infrequent outcome measures. Furthermore, observational studies might provide insight into treatment effects in a more heterogeneous patient population compared with the usually highly selected patient populations in RCTs.^13,14,15,16,17,18^ The addition of observational studies in this meta-analysis could increase sample size and heterogeneity in patient characteristics, which could lead to the evaluation of different age groups, compared with the previous highly selected meta-analyses focusing on the elderly.

The primary aim of this systematic review and meta-analysis was to compare functional, clinical, and radiologic outcomes after operative vs nonoperative treatment of distal radius fractures in adults. As a secondary aim, we sought to compare outcomes in studies that only included patients 60 years or older and other studies that included patients 18 years or older. Finally, we compared effect estimates from RCTs and observational studies.

## Methods

This systematic review and meta-analysis was performed and reported according to the Meta-analysis of Observational Studies in Epidemiology (MOOSE) and the Preferred Reporting Items for Systematic Reviews and Meta-analyses (PRISMA) reporting guidelines.^19,20,21^ This review of the literature did not require approval from the independent ethics committee or institutional review board of the participating institutions.

### Search Strategy and Selection Criteria

The PubMed/MEDLINE, Embase, CENTRAL (Cochrane Central Register of Controlled Trials), and CINAHL (Cumulative Index to Nursing and Allied Health Literature) databases were searched from inception to June 15, 2019, for studies comparing operative vs nonoperative treatment of distal radius fractures by 2 reviewers (Y.O. and J.P.). The search syntax is provided in eTable 1 in the Supplement. Duplicate articles were removed, and 2 reviewers (Y.O. and J.P.) independently performed title and abstract screening for eligibility of identified studies. All published comparative studies, including RCTs and observational studies, reporting on the comparison of operative vs nonoperative treatment of distal radius fractures were eligible for inclusion.

After title and abstract screening, full-text articles were reviewed independently by the same 2 reviewers (Y.O. and J.P.). Inclusion criteria consisted of (1) acute distal radius fracture, (2) operative treatment (internal or external fixation) vs nonoperative treatment (cast immobilization, splinting, or bracing), (3) patients 18 years or older, and (4) reporting of functional outcome. Exclusion criteria consisted of (1) treatment for refracture, (2) language other than English, (3) no availability of full text, and (4) letters, meeting proceedings, and case reports. Disagreements on eligibility of full-text articles were resolved by consensus or by discussion with a third reviewer (M.H.). References of included studies were screened, and backward citation tracking was performed using Web of Science to identify articles not found in the original literature search.

### Data Extraction

Data extraction was performed independently by 2 reviewers (Y.O. and J.P.) with the use of a predefined data extraction form. The following characteristics were extracted from the included studies: first author, year of publication, study design, country in which the study was performed, study period, number of included patients, follow-up period, included age groups, AO fracture classification, operative method, and nonoperative method. Studies reporting on patient cohorts described in previously published articles were excluded or merged.

### Quality Assessment

The methodological quality of included studies was independently assessed by 2 reviewers (Y.O. and J.P.) using the Methodological Index for Non-randomized Studies (MINORS).^22^ The MINORS is a validated instrument for the assessment of methodological quality and clear reporting of nonrandomized surgical studies, resulting in a score ranging from 0 to 24 (higher scores indicate better quality) for comparative studies.^22^ Details on the methodological quality assessment are provided in eTable 2 in the Supplement. Disagreements were resolved by consensus.

### Primary Outcome Measures

The primary outcome measures included medium-term functional outcome measured with the Disabilities of the Arm, Shoulder and Hand questionnaire (DASH) and the overall complication rate after operative and nonoperative treatment. The DASH is a patient-reported outcome instrument developed to measure upper extremity disability and symptoms, resulting in a score ranging from no disability (0) to most severe disability (100).^23^ Functional outcome scores were subdivided according to follow-up as medium term (≤1 year) and long term (>1 year). Complication rate was defined as the overall rate of complications and included reports of infection, nerve injury, chronic pain, complex regional pain syndrome, implant failure, and fracture healing disorders.

### Secondary Outcomes

Secondary functional outcome measures included the Patient-Rated Wrist Evaluation score^24^ and the visual analogue scale score.^25^ Secondary clinical outcome measures included grip strength, range of wrist extension (in degrees), range of wrist flexion (in degrees), range of wrist pronation (in degrees), range of wrist supination (in degrees), radial deviation (in degrees), and ulnar deviation (in degrees). Secondary radiologic outcome measures included volar tilt (in degrees), radial inclination (in degrees), radial height (in millimeters), articular step-off (in millimeters), and ulnar variance (in millimeters).

### Statistical Analysis

Data were analyzed in September 2019. Continuous variables are presented as means with SDs or ranges. Continuous variables were converted to mean (SD) if sufficient information was available, using the methods described in the Cochrane Handbook for Systematic Reviews of Interventions.^26^ Dichotomous variables were extracted as absolute number and percentage. Dichotomous outcomes were pooled using the Mantel-Haenszel method and presented as risk ratios (RRs) with 95% CIs. Continuous outcomes were pooled using the inverse variance weighting method and presented as mean differences (MDs) with 95% CIs.^26^ All analyses were performed using random-effects models. Statistical heterogeneity between studies was assessed by visual inspection of forest plots and by the *I*^2^ and χ^2^ statistics for heterogeneity. The significance level for treatment effects was determined by the overall-effect *z* test. All analyses were performed stratified by study design (RCT or observational study). Differences in effect estimates between the 2 subgroups were assessed, as described in the Cochrane Handbook for Systematic Reviews of Interventions.^26^ The significance level for difference in effect estimates across the subgroups was determined by the test for subgroup differences. The significance level for treatment effects and differences across the subgroups was defined as 2-sided *P* < .05. Potential publication bias was assessed by visual inspection of funnel plots with MD or RR and standard error and Egger statistical tests.^27,28^ Statistical meta-analyses were performed using Review Manager (RevMan, version 5.3.5).^29^ Additional random-effects meta-regression analyses and Egger statistical tests for publication bias were performed in R, version 3.6.1 (R Project for Statistical Computing).^30^

### Subgroup Analyses

Subgroup analyses were performed for the primary outcome measures, the medium-term DASH score and complication rate, by stratifying by studies that only included patients 60 years or older and the other studies that included patients 18 years or older. In addition, random-effects meta-regression was performed, in which the reported mean difference in medium-term DASH score was regressed according to the mean age of the different study populations. Secondary subgroup analyses were performed including only high-quality studies and according to year of the study period. High-quality studies were defined as having a MINORS score of 16 or higher. The subgroup analyses for study period were performed with studies that included patients after 2008 to account for the development of new operative techniques and nonoperative treatment modalities during the past decade.

## Results

### Search

A flowchart of the literature search and study selection is shown in eFigure 1 in the Supplement. In total, 23 unique studies were included in this systematic review and meta-analysis, including 8 RCTs and 15 observational studies.^31,32,33,34,35,36,37,38,39,40,41,42,43,44,45,46,47,48,49,50,51,52,53^

### Study Characteristics

The 23 studies included 2254 unique patients, of whom 1040 were treated operatively and 1214 nonoperatively. The overall weighted mean age was 67 (range, 22-90) years (66 years in the operative group and 67 years in the nonoperative group). Overall, the studies that presented sex data included 425 men (19.4%) and 1769 women (80.6%). The overall follow-up ranged from 6 to 156 months. The baseline characteristics for RCTs and observational studies are presented in Table 1. In addition, eTable 3 in the Supplement presents the treatment and fracture characteristics of all included studies. The studies included 851 patients (37.8%) who sustained an AO fracture type A; 164 (7.3%), type B; 689 (30.6%), type C; and 550 (24.4%), unknown type.

**Table 1.** Baseline Characteristics of Included Studies in a Meta-analysis of Distal Radius Fractures

Study	Study period	Design	Country	No. of participants	Treatment group, No. of participants		Age group, y	Mean age by treatment group, y^a^		Male participants by treatment group, No. (%)^a^		Mean follow-up by treatment group, mo^a^	
					Operative	Nonoperative		Operative	Nonoperative	Operative	Nonoperative	Operative	Nonoperative
**Randomized clinical trials**													
Abbaszadegan et al,^31^ 1990	NA	RCT	Sweden	47	23	24	>18	63 (range, 22-75)		11 (23.4)		12	
Arora et al,^35^ 2011	2005-2008	RCT	Austria	73	36	37	>65	75.9 (range, 65-88)	77.4 (range, 65-89)	8 (22.2)	10 (27.0)	12	
Azzopardi et al,^36^ 2005	1997-2000	RCT	Scotland	54	27	27	>60	72 (SD, 8)	71 (SD, 9)	4 (14.8)	2 (7.4)	12	
Bartl et al,^38^ 2014	2008-2012	RCT	Germany	149	68	81	>65	75.3 (SD, 6.7)	74.4 (SD, 7.1)	9 (13.2)	12 (14.8)	12	
Martinez-Mendez et al,^46^ 2018	2012-2015	RCT	Spain	97	50	47	>60	67 (SD, 8)	70 (SD, 7)	11 (22.0)	10 (21.3)	29 (range, 24-48)	
Mulders et al,^47^ 2019	2013-2016	RCT	The Netherlands	92	48	44	18-75	59 (IQR, 42-66)	60 (IQR, 52-65)	16 (33.3)	7 (15.9)	12	
Sharma et al,^48^ 2014	2009-2010	RCT	India	64	32	32	22-55	52.4 (SD, 9.1)	48.1 (SD, 10.3)	12 (37.5)	14 (43.8)	24	
Sirniö et al,^49^ 2019	2008-2014	RCT	Finland	80	38	42	>50	62 (range, 50-79)	64 (range, 50-82)	1 (2.6)	3 (7.1)	24	
**Observational studies**													
Aktekin et al,^32^ 2010	NA	RCS	Turkey	46	22	24	>65	69.8 (SD, 4.5)	71.2 (SD, 5.2)	9 (40.9)	5 (20.8)	27 (SD, 10.9)	23 (SD, 11.0)
Alm-Paulsen et al,^33^ 2012	1997-2006	RCS	Norway	60	30	30	30-85	61 (range, 37-80)	60 (range, 34-78)	NA	NA	72 (range, 36-84)	84 (range, 36-156)
Arora et al,^34^ 2009	2000-2005	RCS	Austria	114	53	61	>70	75.9 (SD, 4.8)	80.9 (SD, 5.7)	17 (32.1)	19 (31.1)	52 (range, 12-64)	62 (range, 12-81)
Barai et al,^37^ 2018	2014-2015	RCS	New Zealand	116	29	87	>18	58 (IQR, 47-70)	56 (IQR, 29-68)	10 (34.5)	25 (28.7)	18	
Chan et al,^39^ 2014	2009-2010	PCS	Singapore	75	40	35	>65	71.5 (SD, 5.2)	75.8 (SD, 9.3)	6 (15.0)	5 (14.3)	12	
Egol et al,^40^ 2010	2004-2008	RCS	United States	90	44	46	>65	73 (SD, 6.2)	76 (SD, 7.0)	8 (18.2)	6 (13.0)	12	
Gong et al,^41^ 2011	2008-2009	PCS	South Korea	50	26	24	>18	53 (SD, 13)	58 (SD, 13)	6 (23.1)	3 (12.5)	6	
Hung et al,^42^ 2015	2010-2013	RCS	China	57	26	31	61-80	65 (range, 61-80)	64 (range, 61-80)	5 (19.2)	7 (22.6)	12 (range, 6-24)	
Jordan et al,^43^ 2016	2011-2013	RCS	United Kingdom	159	74	85	>50	66.3 (SD, 10.7)	68.7 (SD, 11.8)	12 (16.2)	6 (7.1)	24 (range, 17-36)	
Larouche et al,^44^ 2016	NA	PCS	Canada	129	70	59	>55	64.6 (SD, 7.6)		12 (9.3)		12	
van Leerdam et al,^52^ 2019	2012	RCS	Netherlands	272	87	185	>18	62 (SD, 16)		69 (25.4)		46 (SD, 4)	
Lutz et al,^45^ 2014	1995-2011	RCS	United Kingdom	258	129	129	>65	74 (SD, 5; range, 65-90)		21 (8.1)		11.3 (SD, 9.3)	14.9 (SD, 8.9)
Tan et al,^50^ 2012	2006-2009	RCS	United States	63	31	32	>18	65 (SD, 15)	63 (SD, 18)	2 (6.5)	3 (9.4)	13 (range, 12-17)	14 (range, 11-23)
Toon et al,^51^ 2017	2011-2012	RCS	Singapore	60	32	28	>21	52.1 (range, 23-77)	57.4 (range, 26-79)	14 (43.8)	11 (39.3)	12	
Zengin et al,^53^ 2019	2014-2016	RCS	Turkey	49	25	24	>60	66.6 (SD, 7.4)	68.9 (SD, 8.7)	7 (28.0)	7 (29.2)	16.5 (SD, 3.1)	15.6 (SD, 4.4)

Abbreviations: IQR, interquartile range; NA, not available; PCS, prospective cohort study; RCS, retrospective cohort study.

^a^ For studies that did not present characteristics for treatment groups separately, the numbers presented are for the overall study group, and the cells are merged.

The 8 RCTs^31,35,36,38,46,47,48,49^ included 656 patients (29.1%), of whom 322 were treated operatively and 334 nonoperatively. The weighted mean age was 67 years (67 years in the operative group and 68 years in the nonoperative group). The studies included 130 men (19.8%). The operative method was open reduction and internal fixation with a volar plate in 6 studies,^35,38,46,47,48,49^ external fixation in 1 study,^31^ and percutaneous pinning in 1 study.^36^ The conservative method was cast immobilization in all studies.

The 15 observational studies (3 prospective^39,41,44^ and 12 retrospective^32,33,34,37,40,42,43,45,50,51,52,53^ cohort studies) included 1598 patients (70.9%). Operative treatment was performed in 718 patients (44.9%), and 880 (55.1%) were treated nonoperatively. The weighted mean age in the studies was 67 years (66 years in the operative group and 67 years in the nonoperative group). The studies that presented sex data included 295 men (19.2%). The operative method was open reduction and internal fixation with a volar plate in 6 studies,^34,39,41,42,51,53^ external fixation in 1 study,^32^ percutaneous pinning in 1 study,^33^ intramedullary nail fixation in 1 study,^50^ k-wire fixation in 1 study,^43^ and unclear or a combination of methods in 5 studies.^37,40,44,45,52^ The conservative method was cast immobilization in 13 studies^32,34,37,39,40,41,42,43,44,45,50,51,53^ and unclear in 2 studies.^33,52^

### Quality Assessment

The overall mean MINORS score was 17.2 (SD, 3.6; range, 11-23). The mean MINORS score for the RCTs was 20.9 (SD, 2.0; range, 17-23). The mean MINORS score for the observational studies was 15.2 (SD, 2.5; range, 11-20). The details and distribution of MINORS scores are provided in eTable 4 in the Supplement.

### Primary Outcome Measures

**Figure 1.**
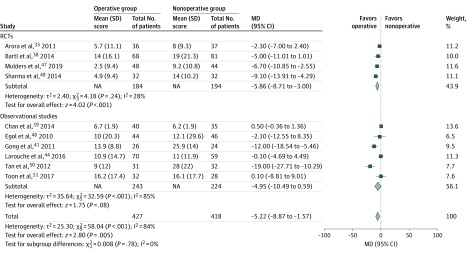
Forest Plot of Medium-Term Disabilities of the Arm, Shoulder and Hand Questionnaire (DASH) Score Medium term indicates 1 year or less. Results are reported using inverse-variance weighted random-effects methods. MD indicates mean difference; RCT, randomized clinical trial. Size of diamond markers indicates weight.

Medium-term (≤1 year) functional outcome assessed according to the DASH score was reported in 10 studies, including 4 RCTs^35,38,47,48^ and 6 observational studies,^39,40,41,44,50,51^ with 845 patients. The AO fracture type was known for 716 patients. Of these, 402 patients (56.1%) sustained an AO fracture type A; 55 (7.7%), type B; and 259 (36.2%), type C. The overall pooled effect revealed that operative treatment was associated with a significant improvement in the medium-term DASH score compared with nonoperative treatment (MD, −5.22 [95% CI, −8.87 to −1.57]; *P* = .005; *I*^2^ = 84%) (Figure 1). There was no difference in effect estimates from RCTs compared with observational studies (test for subgroup differences, χ^2^_1_ = 0.008; *P* = .78). There was no visual asymmetry in the funnel plot (eFigure 2 in the Supplement). The Egger linear regression test (slope, 1.51; *t* = 1.61; *P* = .15) indicated no evidence of publication bias.

**Figure 2.**
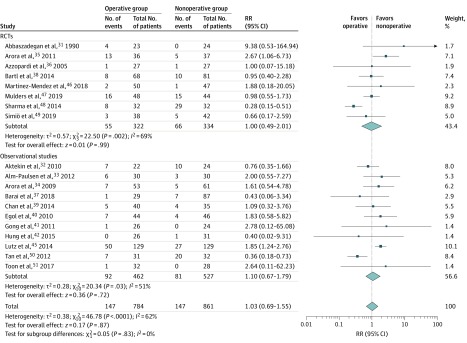
Forest Plot of Complication Rate of Distal Radius Fractures Results are reported using inverse-variance weighted random-effects methods. RCT indicates randomized clinical trial; RR, risk raio. Size of diamond markers indicates weight.

Complication rate was reported in 19 studies, including 8 RCTs^31,35,36,38,46,47,48,49^ and 11 observational studies.^32,33,34,37,39,40,41,42,45,50,51^ The overall pooled effect showed no difference in complication rate between operative and nonoperative treatment with an RR of 1.03 (95% CI, 0.69-1.55; *P* = .87; *I*^2^ = 62%) (Figure 2). No difference was found in effect estimates from RCTs compared with observational studies (test for subgroup differences, χ^2^_1_ = 0.05; *P* = .83). There was no visual asymmetry in the funnel plot (eFigure 3 in the Supplement). The Egger linear regression test (slope, 1.11; *t* = 0.02; *P* = .99) indicated no evidence of publication bias. The incidence of complications was 18.8% (147 of 784) after operative treatment compared with 17.1% (147 of 861) after nonoperative treatment. Complication classification and incidence are presented in Table 2. The main complications after operative treatment were nerve injury or symptoms (26 of 784 [3.3%]) and infection (25 of 784 [3.2%]). The main complications after nonoperative treatment were nerve injury or symptoms (57 of 861 [6.6%]) and chronic pain or complex regional pain syndrome (33 of 861 [3.8%]).

**Table 2.** Complications of Included Studies in a Meta-analysis of Distal Radius Fractures

Complication classification	Incidence, No. (%)	
	Operative group (n = 784)	Nonoperative group (n = 861)
Infection	25 (3.2)	0
Nerve injury or symptoms	26 (3.3)	57 (6.6)
Carpal tunnel syndrome	8 (1.0)	12 (1.4)
Chronic pain or CRPS	21 (2.7)	33 (3.8)
Tendon injury	16 (2.0)	4 (0.5)
Implant failure	2 (0.3)	0
Wound dehiscence	1 (0.1)	0
Tenosynovitis	23 (2.9)	4 (0.5)
Not specified or other	22 (2.8)	14 (1.6)
Malunion, nonunion, or malposition	3 (0.4)	23 (2.7)
Total	147 (18.8)	147 (17.1)

Abbreviation: CRPS, complex regional pain syndrome.

### Secondary Functional Outcome Measures

No difference was found regarding the secondary functional outcome measures (eFigures 4-8 in the Supplement). Descriptive details on functional outcome measures are provided in eTable 5 in the Supplement.

### Secondary Clinical Outcome Measures

Grip strength was reported in 13 studies, including 6 RCTs^35,36,46,47,48,49^ and 7 observational studies,^33,34,39,40,50,51,53^ and was assessed in kilograms (509 patients) and percentage of the unaffected side (462 patients). Both methods revealed an improvement of the grip strength in favor of operative treatment in grip strength measured in kilograms (MD, 2.73 [95% CI, 0.15-5.32]; *P* = .04; *I*^2^ = 79%) and grip strength as a percentage of the unaffected side (MD, 8.21 [95% CI, 2.26-14.15]; *P* = .007; *I*^2^ = 76%) (eFigures 9 and 10 in the Supplement).

There was no difference regarding range of wrist extension, range of wrist flexion, range of wrist pronation, range of wrist supination, radial deviation, and ulnar deviation (eFigures 11-16 in the Supplement). Descriptive details on clinical outcome measures are provided in eTables 6 and 7 in the Supplement.

### Secondary Radiologic Outcome Measures

There was a significant improvement in favor of operative treatment regarding volar tilt (MD, 5.49° [95% CI, 2.94°-8.03°]; *P* < .001; *I*^2^ = 90%), radial inclination (MD, 3.46° [95% CI, 2.73°-4.18°]; *P* = .001; *I*^2^ = 54%), radial height (MD, 2.36 [95% CI, 1.87-2.85] mm; *P* < .001; *I*^2^ = 54%), and articular step-off (MD, −0.27 [95% CI, −0.51 to –0.03] mm; *P* = .03; *I^2^* = 83%) (eFigures 17-20 in the Supplement). There was no difference between treatment groups regarding the ulnar variance (MD, −0.29 [95% CI, −0.97 to 0.40] mm; *P* = .41; *I*^2^ = 92%) (eFigure 21 in the Supplement). Descriptive details on radiologic outcome measures are provided in eTable 8 in the Supplement.

### Subgroup Analyses

The results of the subgroup analyses are presented in Table 3. The medium-term DASH score for studies that only included patients 60 years or older was reported in 4 studies (2 RCTs^35,38^ and 2 observational studies^39,40^), with 387 patients and an overall mean age of 75 years. These studies included 247 patients (63.8%) who sustained an AO fracture type A; 9 (2.3%), type B; and 131 (33.9%), type C. The overall pooled effect showed no difference in the medium-term DASH score (MD, −0.98 [95% CI, −3.52 to 1.57]; *P* = .45; *I*^2^ = 34%) (eFigure 22 in the Supplement). The medium-term DASH score for other studies that included patients 18 years or older was reported in 6 studies (2 RCTs^47,48^ and 4 observational studies^41,44,50,51^), with 458 patients and an overall mean age of 59 years. The AO fracture type was known for 329 patients, including 155 (47.1%) who sustained an AO fracture type A; 46 (14.0%), type B; and 128 (38.9%), type C. The overall pooled effect revealed operative treatment was associated with a significant improvement of the medium-term DASH score compared with nonoperative treatment (MD, −7.50 [95% CI, −12.40 to −2.60]; *P* = .003; *I*^2^ = 77%) (eFigure 22 in the Supplement). There was a significant difference in effect estimates from studies that only included patients 60 years or older compared with the other studies that included patients 18 years or older (test for subgroup differences, χ^2^_1_ = 5.37; *P* = .02) (eFigure 22 in the Supplement).

**Table 3.** Subgroup Analyses of Included Studies in a Meta-analysis of Distal Radius Fractures

Subgroup	Medium-term DASH score				Complication rate			
	No. of studies	MD (95% CI)	*P* value	*I*^2^ statistic, %^a^	No. of studies	RR (95% CI)	*P* value	*I*^2^ statistic, %^a^
All	10	−5.22 (−8.87 to −1.57)	.005	84	19	1.03 (0.69 to 1.55)	.87	62
Studies only age ≥60 y	4	−0.98 (−3.52 to 1.57)	.45	34	10	1.51 (1.15 to 2.00)	.003	0
Other studies age ≥18 y	6	−7.50 (−12.40 to −2.60)	.003	77	9	0.73 (0.39 to 1.38)	.34	60
High-quality studies	7	−6.98 (−11.80 to −2.17)	.004	90	11	0.88 (0.50 to 1.55)	.66	64
Study period 2008 or later	6	−5.31 (−10.20 to −0.43)	.03	87	10	0.72 (0.44 to 1.17)	.18	34

Abbreviations: DASH, Disabilities of the Arm, Shoulder and Hand questionnaire; MD, mean difference; RR, risk ratio.

^a^ Indicates heterogeneity.

**Figure 3.**
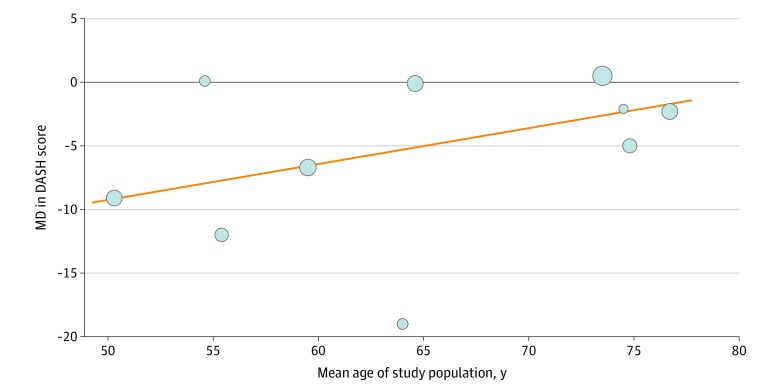
Random-Effects Meta-regression Plot Data are expressed as medium-term (≤1 year) Disabilities of the Arm, Shoulder and Hand questionnaire (DASH) score (operative vs nonoperative groups) according to mean age of the study population in a meta-analysis of distal radius fractures. Circles represent the different studies, with circle size corresponding to the study weight. The black line represents the null value. MD indicates mean difference.

Results of the random-effects meta-regression analysis are shown in Figure 3; the trend of the MD in medium-term DASH score appears to decrease by 0.28 per year increase in the mean age of the study population (estimated regression coefficient, 0.28 [95% CI, −0.03 to 0.59]; *P* = .07). In the studies that only included patients 60 years or older, there was a significant difference in complication rate in favor of nonoperative treatment (RR, 1.51 [95% CI, 1.15-2.00]; *P* = .003; *I*^2^ = 0%), compared with other studies that included patients 18 years or older (RR, 0.73 [95% CI, 0.39-1.38]; *P* = .34; *I*^2^ = 60%) (test for subgroup differences: *P* = .04) (eFigure 23 in the Supplement). The results of all the secondary subgroup analyses are presented in Table 3 and eFigures 24 to 27 in the Supplement.

## Discussion

Operative treatment of distal radius fractures was associated with an improvement in medium-term DASH score compared with nonoperative treatment in adults. No difference was observed in complication rate between treatment groups. There was also an improvement of grip strength in favor of operative treatment. However, no difference was found in medium-term DASH score in the subgroup of studies that only included patients 60 years or older. Furthermore, in the studies that only included these patients, a significant difference in complication rate favored nonoperative treatment. Subgroup analyses with high-quality studies and studies with a study period after 2008 showed similar results, compared with the primary analyses. No difference was found between effect estimates from RCTs and observational studies regarding the primary outcome measures (medium-term DASH score and complication rate).

The pooled effect estimates showed that operative treatment was associated with an improvement in medium-term DASH score compared with nonoperative treatment, which is in contrast to findings of previous meta-analyses.^3,4,5^ Song et al^3^ pooled functional outcome according to the medium-term DASH score at 12 months from 2 studies with 133 patients and found no difference between treatment groups. Ju et al^4^ pooled the DASH score from 6 studies with 577 patients and reported no difference. Chen et al^5^ found no difference in DASH score between treatment groups after they evaluated 7 studies with 600 patients. The present review included 10 studies with 845 patients in the medium-term DASH analysis, which resulted in an increased number of patients available for analyses, thus exceeding the samples of previous meta-analyses. Furthermore, only the meta-analysis by Song et al^3^ evaluated the DASH score at 12 months. The meta-analyses by Ju et al^4^ and Chen et al^5^ did not distinguish between medium-term and long-term DASH scores, including the studies by Arora et al^34^ and Aktekin et al^32^ in their analyses. In the present review, the DASH scores reported by Arora et al^34^ and Aktekin et al^32^ were used for the evaluation of the long-term DASH score owing to their long-term follow-up periods to 81 months. In general, medium-term functional outcome can be assumed to reflect the effect of treatment, with long-term follow-up being influenced by other conditions, events, or patient factors that in turn could influence functional outcome scores. Reports have shown that the DASH score after distal radius fracture treatment tends to plateau after 12 months.^54,55^

The previous meta-analyses have mainly focused on elderly patients. Ju et al^4^ and Chen et al^5^ specifically focused on patient populations 60 years and older. Song et al^3^ included only studies with patients 45 years or older, with most of the patients in their DASH analyses 60 years or older. These findings are in accordance with our subgroup analyses of the studies that only included patients 60 years or older, showing no difference in medium-term DASH score. However, we found a significant improvement in medium-term DASH score in the subgroup of other studies that included patients 18 years or older. To our knowledge, with the analyses of 6 studies with 458 patients, this study is the first meta-analysis to evaluate functional outcome focusing on patient populations 18 years or older. The random-effects meta-regression plot confirmed this trend; however, with only 10 studies and based on the mean age of the complete population, the regression is underpowered. Meta-regression is an extension to subgroup analyses that allows the effect of characteristics to be investigated. However, this is rarely possible owing to inadequate numbers of studies, and meta regression should generally not be considered when there are fewer than 10 studies, as described in the Cochrane Handbook for Systematic Reviews of Interventions.^26^ This trend shows that, to improve personalized care, further evaluation of individual patient data meta-analyses is needed.

We found no difference in the overall complication rate between operative and nonoperative treatment, in accordance with the studies by Song et al^3^ and Yu et al.^56^ However, in our analyses with studies that only included patients 60 years or older, a significant difference favored nonoperative treatment. These findings could indicate that operative treatment results in a higher risk of complications in the elderly population. The study by Chen et al^5^ subdivided complications into minor and major, classifying minor as not requiring surgical treatment. They found no significant difference in minor complications; however, there was a significant difference in major complications, with the most common major complications being nerve and tendon injuries. In the present review, we did not subdivide major and minor complications; however, we did present complication classifications with incidence, showing that nerve injury or symptoms were the main complications in both groups. In the present review, we were not able to accurately compare major and minor complications or specify nerve injuries and symptoms. Unfortunately, this remains difficult owing to limited or missing information regarding the presentation and treatment of complications in studies.

We found a significant improvement of grip strength in favor of operative treatment, which is in contrast with 2 previous meta-analyses. Ju et al^4^ found no significant difference in grip strength in their analysis of 4 studies with 337 patients. Song et al^3^ evaluated grip strength at 12 months with the results of 2 studies with 133 patients and found no difference. However, both the meta-analyses by Ju et al^4^ and Song et al^3^ could be limited by the number of included patients in their grip strength analyses. On the contrary, Chen et al^5^ reported grip strength was significantly greater in the operative group in their analyses of 5 studies with 398 patients. In the present review, grip strength was reported in 13 studies and assessed in kilograms and percentage of the unaffected side with 509 and 462 patients, respectively.

We found no significant difference between treatment groups regarding range of wrist motions. These findings are also in accordance with those of Chen et al,^5^ who reported wrist range of motion did not differ significantly at final follow-up between the 2 treatment groups.

Subgroup analyses including only high-quality studies or studies performed after 2008 showed similar results regarding the primary outcome measures, medium-term DASH score and complication rate, compared with the primary analyses. Furthermore, no difference was observed in effect estimates from RCTs and observational studies regarding the primary outcome measures. These results are in line with previous orthopedic trauma meta-analyses,^9,10,11,12^ including RCTs and observational studies, showing high-quality observational studies to result in similar treatment effects compared with RCTs. Reports^9,11,12,13,14,15,18^ have shown that differences in effect estimates between RCTs and observational studies tend to be small. Randomized clinical trials require strict conditions such as participant selection, inclusion and exclusion criteria, randomization method, and outcome measurements. Patient population in daily clinical practice might differ from the often highly selected patient populations in RCTs.^57,58,59^ The results of observational studies, representing daily clinical practice with various levels of surgical experience and differences in operative techniques, could complement those of RCTs, provided that confounding has been adequately addressed.^17,18^ Including observational studies in meta-analyses that evaluate surgical interventions increases sample size and may facilitate subgroup analysis. These results could help to understand the generalizability of previous results and improve existing guidelines.

Operative treatment of distal radius fractures results in a significant improvement of the medium-term DASH score and grip strength in adults, with no significant difference in overall complication rate. These results might support the international increase of operative treatment of distal radius fractures.^6^ Operative treatment might be the preferred treatment for distal radius fractures in younger patients. However, patient- and fracture-specific factors (patient preference, handedness, occupation, comorbidities, fracture displacement, etc) should always be taken into consideration, and patients should be counseled regarding incidence of complications. Studies have shown an increase of distal radius fractures in patients aged 17 to 64 years.^2^ Hence, future studies should also focus on the nonelderly population, because traditionally most studies on this topic solely include patient populations 60 years or older. Further investigation is warranted to understand the optimal treatment methods and outcomes in this nonelderly, generally healthy, and still working age group. Furthermore, for the evaluation of the effect on the health and well-being of nonelderly adults, future studies could also focus on return to sporting activity and return to work, aside from traditional outcomes. Unfortunately, comparison of literature remains difficult owing to a wide variety of AO fracture types, different age groups, operative treatments, the use of different functional outcome measures, and duration of follow-up. Further research is needed for the development of patient- and fracture-specific guidelines.

### Limitations

Potential limitations in this review need to be acknowledged. First, analyses could be influenced by missing results; however, an extensive electronic database search was performed, and funnel plots did not indicate evidence of publication bias. Second, the subgroup analyses regarding age were stratified based on the inclusion criteria of studies, which resulted in overlap of the age distributions between the subgroup analyses. Nevertheless, there still was a substantial difference in the overall mean age in both subgroups (59 years vs 75 years). Furthermore, it should be noted that the cutoff of 60 years or older is arbitrarily chosen to compare our findings with the previous meta-analyses that mainly focused on patient populations 60 years and older. We acknowledge that better evidence is lacking, and further evaluation using individual patient data meta-analysis is needed. Third, we were not able to accurately classify all complications. Unfortunately, this remains difficult owing to insufficient or missing information. In addition, this review included a variety of fracture types. The AO fracture types A, B, and C seemed equally distributed throughout the different functional outcome analyses, with most studies including AO types A and C fractures. However, reports have shown patient-reported outcomes to vary in the setting of multiple-trauma or high-energy injury mechanisms. In addition to demographic and fracture characteristics, factors related to injury context (multiple-trauma, high-energy mechanism) could also account for differences in patient-reported wrist function after distal radius fractures.^60,61^

## Conclusions

This meta-analysis found that operative treatment of distal radius fractures improved the medium-term DASH score and grip strength compared with nonoperative treatment in adults. There was no difference in complication rate between treatment groups. However, there was no difference in medium-term DASH score in the subgroup of studies that only included patients 60 years or older. Furthermore, in this subgroup, operative treatment resulted in a significantly higher complication rate. Our findings suggest that operative treatment might be more effective and have a greater effect on the health and well-being of younger, nonelderly patients. However, to improve personalized care, this trend needs to be confirmed with patient-level data. Further evaluation of individual patient data meta-analyses is needed.

## Notes

Supplement. **eTable 1.** Search Syntax Performed Last on June 15, 2019 **eTable 2.** Quality Assessment According to the MINORS Criteria in a Meta-analysis of Distal Radius Fractures **eTable 3.** Treatment Characteristics of Included Studies in a Meta-analysis of Distal Radius Fractures **eTable 4. **Quality Assessment of Included Studies in a Meta-analysis of Distal Radius Fractures **eTable 5. **Functional Outcome Measures of Included Studies in a Meta-analysis of Distal Radius Fractures **eTable 6. **Clinical Outcome Measures of Included Studies in a Meta-analysis of Distal Radius Fractures **eTable 7. **Clinical Outcome Measures of Included Studies in a Meta-analysis of Distal Radius Fractures **eTable 8. **Radiologic Outcome Measures of Included Studies in a Meta-analysis of Distal Radius Fractures **eFigure 1.** PRISMA Flow Diagram Representing the Search and Selection of Studies Comparing Operative vs Nonoperative Treatment of Distal Radius Fractures **eFigure 2.** Funnel Plot of Medium-Term (≤1 y) DASH Score in a Meta-analysis of Distal Radius Fractures **eFigure 3. **Funnel Plot of Complication Rate in a Meta-analysis of Distal Radius Fractures **eFigure 4. **Forest Plot of Long-Term (>1 y) DASH Score in a Meta-analysis of Distal Radius Fractures **eFigure 5. **Forest Plot of Medium-Term (≤1 y) PRWE Score in a Meta-analysis of Distal Radius Fractures **eFigure 6. **Forest Plot of Long-Term (>1 y) PRWE Score in a Meta-analysis of Distal Radius Fractures **eFigure 7. **Forest Plot of Medium-Term (≤1 y) VAS Score in a Meta-analysis of Distal Radius Fractures **eFigure 8. **Forest Plot of Long-Term (>1 y) VAS Score in a Meta-analysis of Distal Radius Fractures **eFigure 9. **Forest Plot of Grip Strength in Kilograms in a Meta-analysis of Distal Radius Fractures **eFigure 10.** Forest Plot of Grip Strength as Percentage of Unaffected Side in a Meta-analysis of Distal Radius Fractures **eFigure 11. **Forest Plot of Range of Wrist Extension in Degrees in a Meta-analysis of Distal Radius Fractures **eFigure 12. **Forest Plot of Range of Wrist Flexion in Degrees in a Meta-analysis of Distal Radius Fractures **eFigure 13. **Forest Plot of Range of Wrist Pronation in Degrees in a Meta-analysis of Distal Radius Fractures **eFigure 14. **Forest Plot of Range of Wrist Supination in Degrees in a Meta-analysis of Distal Radius Fractures **eFigure 15. **Forest Plot of Radial Deviation in Degrees in a Meta-analysis of Distal Radius Fractures **eFigure 16. **Forest Plot of Ulnar Deviation in Degrees in a Meta-analysis of Distal Radius Fractures **eFigure 17. **Forest Plot of Volar Tilt in Degrees in a Meta-analysis of Distal Radius Fractures **eFigure 18. **Forest Plot of Radial Inclination in Degrees in a Meta-analysis of Distal Radius Fractures **eFigure 19. **Forest Plot of Radial Height in Millimeters in a Meta-analysis of Distal Radius Fractures **eFigure 20. **Forest Plot Of Articular Step-off in Millimeters in a Meta-analysis of Distal Radius Fractures **eFigure 21. **Forest Plot of Ulnar Variance in Millimeters in a Meta-analysis of Distal Radius Fractures **eFigure 22. **Forest Plot of Medium-Term (≤1 y) DASH Score for Studies That Only Included Patients With Age >60 Years and Other Studies That Included Patients With Age ≥18 Years in a Meta-analysis of Distal Radius Fractures **eFigure 23. **Forest Plot of Complication Rate for Studies That Only Included Patients With Age >60 Years and Other Studies That Included Patients With Age ≥18 Years in a Meta-analysis of Distal Radius Fractures **eFigure 24. **Forest Plot of Medium-Term (≤1 y) DASH Score in High-Quality Studies in a Meta-analysis of Distal Radius Fractures **eFigure 25. **Forest Plot of Complication Rate in High-Quality Studies in a Meta-analysis of Distal Radius Fractures **eFigure 26. **Forest Plot of Medium-Term (≤1 y) DASH Score in Studies With a Study Period After 2008 in a Meta-analysis of Distal Radius Fractures **eFigure 27. **Forest Plot of Complication Rate in Studies With a Study Period After 2008 in a Meta-analysis of Distal Radius Fractures
