# Supplementary material for: Operative vs Nonoperative Treatment of Distal Radius Fractures in Adults: A Systematic Review and Meta-analysis
Source: JAMA Netw Open. 2020 Apr 23;3(4):e203497. doi: 10.1001/jamanetworkopen.2020.3497 (PMC7180423; doi:10.1001/jamanetworkopen.2020.3497)
Supplement: Supplement. — eTable 1. Search Syntax Performed Last on June 15, 2019 eTable 2. Quality Assessment According to the MINORS Criteria in a Meta-analysis of Distal Radius Fractures eTable 3. Treatment Characteristics of Included Studies in a Meta-analysis of Distal Radius Fractures eTable 4. Quality Assessment of Included Studies in a Meta-analysis of Distal Radius Fractures eTable 5. Functional Outcome Measures of Included Studies in a Meta-analysis of Distal Radius Fractures eTable 6. Clinical Outcome Measures of Included Studies in a Meta-analysis of Distal Radius Fractures eTable 7. Clinical Outcome Measures of Included Studies in a Meta-analysis of Distal Radius Fractures eTable 8. Radiologic Outcome Measures of Included Studies in a Meta-analysis of Distal Radius Fractures eFigure 1. PRISMA Flow Diagram Representing the Search and Selection of Studies Comparing Operative vs Nonoperative Treatment of Distal Radius Fractures eFigure 2. Funnel Plot of Medium-Term (≤1 y) DASH Score in a Meta-analysis of Distal Radius Fractures eFigure 3. Funnel Plot of Complication Rate in a Meta-analysis of Distal Radius Fractures eFigure 4. Forest Plot of Long-Term (>1 y) DASH Score in a Meta-analysis of Distal Radius Fractures eFigure 5. Forest Plot of Medium-Term (≤1 y) PRWE Score in a Meta-analysis of Distal Radius Fractures eFigure 6. Forest Plot of Long-Term (>1 y) PRWE Score in a Meta-analysis of Distal Radius Fractures eFigure 7. Forest Plot of Medium-Term (≤1 y) VAS Score in a Meta-analysis of Distal Radius Fractures eFigure 8. Forest Plot of Long-Term (>1 y) VAS Score in a Meta-analysis of Distal Radius Fractures eFigure 9. Forest Plot of Grip Strength in Kilograms in a Meta-analysis of Distal Radius Fractures eFigure 10. Forest Plot of Grip Strength as Percentage of Unaffected Side in a Meta-analysis of Distal Radius Fractures eFigure 11. Forest Plot of Range of Wrist Extension in Degrees in a Meta-analysis of Distal Radius Fractures eFigure 12. Forest Plot of Range of Wrist Flexion in [file jamanetwopen-3-e203497-s001.pdf]

Supplementary Online Content

Ochen Y, Peek J, van der Velde D, et al. Operative vs nonoperative treatment of distal radius fractures in adults: a systematic review and meta-analysis. *JAMA Netw Open*. 2020;3(4):e203497.
doi:10.1001/jamanetworkopen.2020.3497

eTable 1. Search Syntax Performed Last on June 15, 2019

eTable 2. Quality Assessment According to the MINORS Criteria in a Meta-analysis of Distal Radius Fractures

eTable 3. Treatment Characteristics of Included Studies in a Meta-analysis of Distal Radius Fractures

eTable 4. Quality Assessment of Included Studies in a Meta-analysis of Distal Radius Fractures

eTable 5. Functional Outcome Measures of Included Studies in a Meta-analysis of Distal Radius Fractures

eTable 6. Clinical Outcome Measures of Included Studies in a Meta-analysis of Distal Radius Fractures

eTable 7. Clinical Outcome Measures of Included Studies in a Meta-analysis of Distal Radius Fractures

eTable 8. Radiologic Outcome Measures of Included Studies in a Meta-analysis of Distal Radius Fractures

eFigure 1. PRISMA Flow Diagram Representing the Search and Selection of Studies Comparing Operative vs Nonoperative Treatment of Distal Radius Fractures

eFigure 2. Funnel Plot of Medium-Term (≤ 1 y) DASH Score in a Meta-analysis of Distal Radius Fractures

eFigure 3. Funnel Plot of Complication Rate in a Meta-analysis of Distal Radius Fractures

eFigure 4. Forest Plot of Long-Term (> 1 y) DASH Score in a Meta-analysis of Distal Radius Fractures

eFigure 5. Forest Plot of Medium-Term (≤ 1 y) PRWE Score in a Meta-analysis of Distal Radius Fractures

eFigure 6. Forest Plot of Long-Term (>1 y) PRWE Score in a Meta-analysis of Distal Radius Fractures

eFigure 7. Forest Plot of Medium-Term (≤1 y) VAS Score in a Meta-analysis of Distal Radius Fractures

eFigure 8. Forest Plot of Long-Term (>1 y) VAS Score in a Meta-analysis of Distal Radius Fractures

eFigure 9. Forest Plot of Grip Strength in Kilograms in a Meta-analysis of Distal Radius Fractures

©2020 Ochen Y et al. *JAMA Network Open*.

eFigure 10. Forest Plot of Grip Strength as Percentage of Unaffected Side in a Meta-analysis of Distal Radius Fractures

eFigure 11. Forest Plot of Range of Wrist Extension in Degrees in a Meta-analysis of Distal Radius Fractures

eFigure 12. Forest Plot of Range of Wrist Flexion in Degrees in a Meta-analysis of Distal Radius Fractures

eFigure 13. Forest Plot of Range of Wrist Pronation in Degrees in a Meta-analysis of Distal Radius Fractures

eFigure 14. Forest Plot of Range of Wrist Supination in Degrees in a Meta-analysis of Distal Radius Fractures

eFigure 15. Forest Plot of Radial Deviation in Degrees in a Meta-analysis of Distal Radius Fractures

eFigure 16. Forest Plot of Ulnar Deviation in Degrees in a Meta-analysis of Distal Radius Fractures

eFigure 17. Forest Plot of Volar Tilt in Degrees in a Meta-analysis of Distal Radius Fractures

eFigure 18. Forest Plot of Radial Inclination in Degrees in a Meta-analysis of Distal Radius Fractures

eFigure 19. Forest Plot of Radial Height in Millimeters in a Meta-analysis of Distal Radius Fractures

eFigure 20. Forest Plot Of Articular Step-off in Millimeters in a Meta-analysis of Distal Radius Fractures

eFigure 21. Forest Plot of Ulnar Variance in Millimeters in a Meta-analysis of Distal Radius Fractures

eFigure 22. Forest Plot of Medium-Term (≤1 y) DASH Score for Studies That Only Included Patients With Age >60 Years and Other Studies That Included Patients With Age ≥18 Years in a Meta-analysis of Distal Radius Fractures

eFigure 23. Forest Plot of Complication Rate for Studies That Only Included Patients With Age >60 Years and Other Studies That Included Patients With Age ≥18 Years in a Meta-analysis of Distal Radius Fractures

eFigure 24. Forest Plot of Medium-Term (≤1 y) DASH Score in High-Quality Studies in a Meta-analysis of Distal Radius Fractures

eFigure 25. Forest Plot of Complication Rate in High-Quality Studies in a Meta-analysis of Distal Radius Fractures

eFigure 26. Forest Plot of Medium-Term (≤1 y) DASH Score in Studies With a Study Period After 2008 in a Meta-analysis of Distal Radius Fractures

eFigure 27. Forest Plot of Complication Rate in Studies With a Study Period After 2008 in a Meta-analysis of Distal Radius Fractures

This supplementary material has been provided by the authors to give readers additional information about their work.

©2020 Ochen Y et al. *JAMA Network Open*.

eTable 1. Search Syntax Performed Last on June 15, 2019	
**Database**	**Syntax**
**PubMed/MEDLINE (n= 1838)**	(((((((((radius fractures[MeSH Terms]) AND distal[Title/Abstract])) OR colles' fracture[MeSH Terms]) OR wrist injuries[MeSH Terms])) OR ((((((((radius[Title/Abstract]) OR radial[Title/Abstract])) AND distal[Title/Abstract])) AND fractur*[Title/Abstract])) OR ((((colles[Title/Abstract]) OR smith[Title/Abstract]) OR barton[Title/Abstract]) OR wrist[Title/Abstract]))) AND fractur*[Title/Abstract])))))) AND ((((((((surgical procedure, operative[MeSH Terms]) OR fracture fixation[MeSH Terms]) OR orthopedic procedure[MeSH Terms]) OR orthopedics[MeSH Terms])) OR ((((((((((((surg*[Title/Abstract]) OR operat*[Title/Abstract]) OR orthop*[Title/Abstract]) OR pin*[Title/Abstract]) OR nail*[Title/Abstract]) OR screw*[Title/Abstract]) OR plat*[Title/Abstract]) OR rod*[Title/Abstract]) OR wire*[Title/Abstract]) OR fix*[Title/Abstract]) OR ORIF[Title/Abstract]) OR ExFix[Title/Abstract])))) AND ((((conservative treatment[MeSH Terms]) OR physical therapy modalities[MeSH Terms])) OR ((((((((((((((((conserv*[Title/Abstract]) OR conven*[Title/Abstract]) OR non-operat*[Title/Abstract]) OR "non operative"[Title/Abstract]) OR nonoperat*[Title/Abstract]) OR non-surg*[Title/Abstract]) OR "non surgical"[Title/Abstract]) OR nonsurg*[Title/Abstract]) OR cast*[Title/Abstract]) OR splint*[Title/Abstract]) OR brace*[Title/Abstract]) OR bracing[Title/Abstract]) OR plaster[Title/Abstract]) OR bandage*[Title/Abstract]) OR tape*[Title/Abstract]) OR taping[Title/Abstract])))
**Embase (n= 1713)**	('distal radius fracture'/exp OR 'colles fracture'/exp OR (('radius':ab,ti OR 'radial':ab,ti) AND 'distal':ab,ti AND 'fractur*':ab,ti) OR (('colles':ab,ti OR 'smith':ab,ti OR 'barton':ab,ti OR 'wrist':ab,ti) AND 'fractur*':ab,ti)) AND ('surgery'/de OR 'orthopedic surgery'/de OR 'surg*':ab,ti OR 'operat*':ab,ti OR 'orthop*':ab,ti OR 'pin*':ab,ti OR 'nail*':ab,ti OR 'screw*':ab,ti OR 'plate*':ab,ti OR 'rod*':ab,ti OR 'wire*':ab,ti OR 'fix*':ab,ti OR 'orif':ab,ti OR 'exfix':ab,ti) AND ('conservative treatment'/de OR 'conservative':ab,ti OR 'conventional':ab,ti OR 'non-operative':ab,ti OR 'non operative':ab,ti OR 'nonoperative':ab,ti OR 'non-surgical':ab,ti OR 'non surgical':ab,ti OR 'nonsurgical':ab,ti OR 'cast*':ab,ti OR 'splint*':ab,ti OR 'brace*':ab,ti OR 'bracing':ab,ti OR 'plaster*':ab,ti OR 'bandage':ab,ti OR 'tape*':ab,ti OR 'taping*':ab,ti)
**CENTRAL (n= 837)**	Radius AND distal AND fracture
**CINAHL (n= 272)**	(((MH distal radius OR TI distal radius OR AB distal radius OR TI radius OR AB radius OR TI radial OR AB radial OR TI colles OR AB colles OR TI smith OR AB smith OR TI barton OR AB barton OR TI wrist OR AB wrist) AND (MH fracture OR MH fractures OR TI fractur* OR AB fractur*)) AND ((MH surgical procedures, operative OR MH orthopedics OR TI surg* OR AB surg* OR TI operat* OR AB operat* OR TI orthop* OR AB orthop* OR TI pin* OR AB pin* OR TI nail* OR AB nail* OR TI screw* OR AB screw* OR TI plate* OR AB plate* OR TI rod* OR AB rod* OR TI wire* OR AB wire* OR TI fix* OR AB fix* OR TI ORIF OR AB ORIF OR TI ExFix OR AB ExFix) AND (MH Conservative Treatment OR MH physical therapy modalities OR TI conservative OR AB conservative OR TI conventional OR AB conventional OR TI non-operative OR AB non-operative OR TI non operative OR AB non operative OR TI nonoperative OR AB nonoperative OR TI non-surgical OR AB non-surgical OR TI non surgical OR AB non surgical OR TI nonsurgical OR AB nonsurgical OR TI cast* OR AB cast* OR TI brace* OR AB brace* OR TI splint* OR AB splint* OR TI bracing OR AB bracing OR TI bandage* OR AB bandage* OR TI tape* OR AB tape* OR TI taping OR AB taping OR TI plaster* OR AB plaster*))

©2020 Ochen Y et al. *JAMA Network Open*.

**eTable 2**. Quality Assessment According to the MINORS Criteria in a Meta-analysis of Distal Radius Fractures			
**Criteria**	**Reported and adequate (2)**	**Reported but inadequate (1)**	**Not reported (0)**
Clearly stated aim	Aim including outcomes reported	Aim reported without outcomes	Not reported
Inclusion consecutive patients	Inclusion/exclusion criteria reported	Unclear description inclusion/exclusion criteria	Not reported
Prospective collection data	Prospective	Not applicable	Not applicable
Appropriate endpoints	Appropriate endpoints to aim study	Endpoints not appropriate to aim study	Not reported
Unbiased assessment	Blinded evaluation of outcomes	Reason not blinding stated	Not reported
Appropriate follow-up	≥ 1 year	< 1 year	Not reported
Loss to follow-up < 5%	≤ 5%	> 5%	Not applicable
Prospective calculation study size	Prospective power-analysis performed	Prospective calculation without power-analysis	Not applicable
Adequate control group	Operative versus nonoperative treatment	Not applicable	Not applicable
Contemporary groups	Study/control group managed during same period	Study/control not managed during same period	Not reported
Baseline equivalence groups	Baseline characteristics described and comparable	Baseline characteristics not comparable	Not reported
Adequate statistical analyses	Statistical analysis described including type of analyses	Inadequate description statistical analysis	Not reported
Items are scored 0 (not reported), 1 (reported but inadequate) or 2 (reported and adequate). The overall score ranging from 0 to 24 for comparative studies			

©2020 Ochen Y et al. *JAMA Network Open*.

**eTable 3.** Treatment Characteristics of Included Studies in a Meta-analysis of Distal Radius Fractures										
**Study**	**Year**	**Overall Number**	**Number**		**Included age group (years)**	**Fracture type (AO), n (%)**			**Operative Treatment**	**Nonoperative Treatment**
			**OP**	**NON**		**A**	**B**	**C**		
**RCTs**										
Abbaszadegan et al.	1990	47	23	24	>18	N/A	N/A	N/A	External fixation	Cast
Arora et al.	2011	73	36	37	>65	22 (30)	0	51 (70)	ORIF volar locking plate	Cast
Azzopardi et al.	2005	54	27	27	>60	54 (100)	0	0	Percutaneous pinning	Cast
Bartl et al.	2014	149	68	81	>65	149 (100)	0	0	ORIF volar locking plate	Cast
Martinez-Mendez et al.	2018	97	50	47	>60	0	0	97 (100)	ORIF volar locking plate	Cast
Mulders et al.	2019	92	48	44	18-75	92 (100)	0	0	ORIF volar locking plate	Cast
Sharma et al.	2014	64	32	32	22-55	0	28 (44)	36 (56)	ORIF volar locking plate	Cast
Sirniö et al.	2019	80	38	42	>50	48 (60)	0	32 (40)	ORIF volar locking plate	Cast
**Observational studies**										
Aktekin et al.	2010	46	22	24	>65	22 (48)	0	24 (52)	External fixation	Cast
Alm-Paulsen et al.	2012	60	30	30	30-85	34 (57)	0	26 (43)	Percutaneous pinning	N/A
Arora et al.	2009	114	53	61	>70	59 (52)	0	55 (48)	ORIF volar locking plate	Cast
Barai et al.	2018	116	29	87	>18	N/A	N/A	N/A	ORIF	Cast
Chan et al.	2014	75	40	35	>65	33 (44)	0	42 (56)	ORIF volar locking plate	Cast
Egol et al.	2010	90	44	46	>65	43 (48)	9 (10)	38 (42)	Volar plate/external fixation	Cast
Gong et al.	2011	50	26	24	>18	26 (52)	0	24 (48)	ORIF volar locking plate	Cast
Hung et al.	2015	57	26	31	61-80	23 (40)	20 (35)	14 (25)	ORIF volar locking plate	Cast
Jordan et al.	2016	159	74	85	>50	102 (64)	3 (2)	54 (34)	K-wire fixation + cast	Cast
Larouche et al.	2016	129	70	59	>55	N/A	N/A	N/A	ORIF	Cast
Leerdam et al.	2019	272	87	185	>18	107 (39)	86 (32)	79 (29)	N/A	N/A
Lutz et al.	2014	258	129	129	>65	N/A	N/A	N/A	Multiple	Cast
Tan et al.	2012	63	31	32	>18	37 (59)	2 (3)	24 (38)	Intramedullary nail fixation	Cast
Toon et al.	2017	60	32	28	>21	0	16 (27)	44 (73)	ORIF volar locking plate	Cast
Zengin et al.	2019	49	25	24	>60	0	0	49 (100)	ORIF volar locking plate	Cast
OP|NON operative/nonoperative; N/A not available; n number; ORIF Open Reduction Internal Fixation; AO Classification of Fractures										

©2020 Ochen Y et al. *JAMA Network Open*.

**eTable 4.** Quality Assessment of Included Studies in a Meta-analysis of Distal Radius Fractures																									
**MINORS criteria**	**RCTs**	Abbaszadegan et al. 1190	Arora et al. 2011	Azzopardi et al. 2005	Bartl et al. 2014	Martinez-Mendez et al. 2018	Mulders et al.2019	Sharma et al. 2014	Sirniö et al. 20119	**Observational studies**	Aktekin et al. 2010	Alm-Paulsen et al. 2012	Arora et al. 2009	Barai et al. 2018	Chan et al. 2014	Egol et al. 2010	Gong et al. 2011	Hung et al. 2015	Jordan et al. 2016	Larouche et al. 2016	Leerdam et al. 2019	Lutz et al. 2014	Tan et al. 2012	Toon et al. 2017	Zengin et al. 2019
Clearly stated aim		1	2	2	2	2	2	2	2		2	2	2	2	2	2	1	2	2	2	2	2	2	2	2
Inclusion of consecutive patients		2	2	2	2	2	2	2	2		2	1	2	2	2	1	2	2	2	1	1	1	2	2	2
Prospective collection of data		2	2	2	2	2	2	2	2		1	1	1	1	2	1	2	0	1	2	0	1	1	1	0
Appropriate endpoints		2	2	2	2	2	2	2	2		2	2	2	2	2	2	2	1	2	2	2	2	2	2	2
Unbiased assessment endpoints		0	1	1	2	1	1	0	0		1	0	1	0	0	0	1	0	2	0	0	0	1	0	0
Appropriate follow-up		2	2	2	2	2	2	2	2		2	2	2	1	2	2	1	1	2	2	2	1	2	2	2
Loss to follow-up < 5%		2	1	1	1	2	2	2	1		1	2	1	1	2	1	1	0	1	0	0	2	2	1	0
Prospective calculation study size		0	2	2	0	2	2	0	2		0	0	0	2	0	0	2	0	0	2	0	0	0	2	0
Adequate control group		2	2	2	2	2	2	2	2		2	2	2	2	2	2	2	2	2	2	0	2	2	2	2
Contemporary groups		2	2	2	2	2	2	2	2		0	1	0	0	2	2	2	2	2	0	2	2	2	0	2
Baseline equivalence of groups		0	2	1	2	2	2	2	1		2	1	2	2	1	2	2	2	2	0	0	1	2	2	2
Adequate statistical analysis		2	2	2	2	2	2	2	2		2	2	2	2	2	2	2	2	2	2	2	2	2	2	2
**Total MINORS score**		**17**	**22**	**21**	**21**	**23**	**23**	**20**	**20**		**17**	**16**	**17**	**17**	**19**	**17**	**20**	**14**	**20**	**15**	**11**	**16**	**20**	**18**	**16**
Items are scored 0 (not reported/ not applicable), 1 (reported but inadequate) or 2 (reported and adequate). The overall score ranging from 0 to 24 for comparative studies																									

©2020 Ochen Y et al. *JAMA Network Open*.

eTable 5. Functional Outcome Measures of Included Studies in a Meta-analysis of Distal Radius Fractures												
**Study**	**DASH ≤ 1 year#**		**PRWE ≤ 1 year#**		**VAS ≤ 1 year#**		**DASH > 1 year#**		**PRWE > 1 year#**		**VAS > 1 year#**	
	**OP**	**NON**	**OP**	**NON**	**OP**	**NON**	**OP**	**NON**	**OP**	**NON**	**OP**	**NON**
**RCTs**												
Abbaszadegan et al. 1990	N/A		N/A		0	1	N/A		N/A		N/A	
Arora et al. 2011	5,7 (11,1)	8,0 (9,3)	12,8 (23,2)	14,6 (22,8)	0,1 (0,3)	0,1 (0,5)	N/A		N/A		N/A	
Azzopardi et al. 2005	N/A		N/A		0,7 (1,3)	1,2 (1,6)	N/A		N/A		N/A	
Bartl et al. 2014	14 (16,1)	19 (21,3)	N/A		N/A		N/A		N/A		N/A	
Martinez-Mendez et al. 2018	N/A		N/A		N/A		16 (14)	28 (21)	17 (13)	30 (25)	2 (2)	3 (2)
Mulders et al. 2019	2,5 (9,4)*	9,2 (10,8)*	4,0 (9,3)*	8,0 (11,9)*	N/A		N/A		N/A		N/A	
Sharma et al. 2014	4,9 (9,4)	14,0 (10,2)	N/A		N/A		N/A		N/A		N/A	
Sirniö et al. 2019	7,2	14,4	N/A		N/A		N/A		N/A		N/A	
**Observational studies**												
Aktekin et al. 2010	N/A		N/A		N/A		21,9 (21,7)	20,3 (13)	N/A		N/A	
Alm-Paulsen et al. 2012	N/A		N/A		N/A		13,5 (15)	10 (13)	11 (15)	8 (12)	5 (11)	2 (7)
Arora et al. 2009	N/A		N/A		N/A		11,1 (12,9)*	11,6 (13,4)*	9,3 (9,3)*	16,9 (12,1)*	1,7 (1,4)	0,7 (1,4)
Barai et al. 2018	N/A		N/A		N/A		12,1 (14,4)	6 (10,9)	N/A		N/A	
Chan et al. 2014	6,7 (1,9)	6,2 (1,9)	N/A		N/A		N/A		N/A		N/A	
Egol et al. 2010	10 (20,3)	12,1 (29,6)	N/A		1,2 (1,7)	1,5 (2,1)	N/A		N/A		N/A	
Gong et al. 2011	13,9 (8,8)	25,9 (14,0)	N/A		N/A		N/A		N/A		N/A	
Hung et al. 2015	N/A		N/A		N/A		4,5	13,6	N/A		N/A	
Jordan et al. 2016	N/A		N/A		N/A		26,6 (6,5)	27,1 (7,7)	N/A		N/A	
Larouche et al. 2016	10,9 (14,7)	11,0 (11,9)	12,3 (15,8)	10,9 (14,1)	N/A		N/A		N/A		N/A	
Leerdam et al. 2019	N/A		N/A		N/A		N/A		17 (22)	8 (15)	N/A	
Lutz et al. 2014	N/A		N/A		N/A		N/A		17 (23)	16 (18)	N/A	
Tan et al. 2012	9 (12)	28 (22)	N/A		N/A		7 (9)	25 (24)	N/A		N/A	
Toon et al. 2017	16,2 (17,4)	16,1 (17,7)	N/A		1,8 (1,6)	1,1 (1,1)	N/A		N/A		N/A	
Zengin et al. 2019	N/A		N/A		N/A		11,7 (8)	17,6 (14,2)	N/A		N/A	
OP|NON operative/nonoperative; n number; # mean (SD); N/A not available; * median												

©2020 Ochen Y et al. *JAMA Network Open*.

**eTable 6.** Clinical Outcome Measures of Included Studies in a Meta-analysis of Distal Radius Fractures								
**Study**	**Complication**$^{\$}$		**Grip strength (kg)**$^{\#}$		**Range wrist extension (°)**$^{\#}$		**Range wrist flexion (°)**$^{\#}$	
	**OP**	**NON**	**OP**	**NON**	**OP**	**NON**	**OP**	**NON**
**RCTs**								
Abbaszadegan et al. 1990	4 (17)	0	N/A		N/A		N/A	
Arora et al. 2011	13 (36)	5 (14)	22,2 (6,3)	18,8 (5,8)	59 (10)	61 (7)	55 (11)	57 (10)
Azzopardi et al. 2005	1 (4)	1 (4)	77 (21)*	72 (17)*	94 (11)*	95 (9)*	87 (12)*	82 (15)*
Bartl et al. 2014	8 (12)	10 (12)	N/A		7,5 (11,7)**	7,5 (10)**	8,2 (11,9)**	11,5 (12,8)**
Martinez-Mendez et al. 2018	2 (4)	1 (2)	73 (27)*	64 (33)*	57 (11)	54 (13)	54 (13)	60 (16)
Mulders et al. 2019	16 (33)	25 (57)	26 (1,9)^	20 (8,4)^	85 (7,4)^	80 (14,8)^	80 (11,9)^	70 (14,8)^
Sharma et al. 2014	8 (25)	29 (91)	89,0 (4,3)*	72,1 (4,4)*	84,3 (2,4)	69,0 (3,91)	83,8 (2,9)	65,9 (7,5)
Sirniö et al. 2019	3 (8)	5 (12)	27 (5)	26 (7)	69 (6)	68 (7)	71 (7)	64 (11)
**Observational studies**								
Aktekin et al. 2010	7 (32)	10 (42)	19	18	63	44	61	53
Alm-Paulsen et al. 2012	6 (20)	3 (10)	30 (12)	27 (10)	N/A		N/A	
Arora et al. 2009	7 (13)	5 (8)	19,4 (6)	21,1 (7)	57 (11,6)	59,8 (7,0)	44,6 (10,4)	49,6 (9,8)
Barai et al. 2018	1 (3)	7 (8)	N/A		N/A		N/A	
Chan et al. 2014	5 (13)	4 (11)	82 (19)*	77 (17)*	N/A		N/A	
Egol et al. 2010	7 (16)	4 (9)	17,7 (7,3)	12,7 (6,5)	54,8 (18,7)	54,6 (14,9)	47,8 (13,1)	51,8 (11,1)
Gong et al. 2011	1 (4)	0	N/A		N/A		N/A	
Hung et al. 2015	0	1 (3)	N/A		60^	60^	60^	60^
Jordan et al. 2016	N/A		N/A		N/A		N/A	
Larouche et al. 2016	14 (11)		N/A		N/A		N/A	
Leerdam et al. 2019	N/A		N/A		N/A		N/A	
Lutz et al. 2014	50 (39)	27 (21)	N/A		N/A		N/A	
Tan et al. 2012	7 (23)	20 (63)	83 (17)*	78 (24)*	N/A		N/A	
Toon et al. 2017	1 (3)	0	83,3 (14,1)*	81,3 (22,9)*	67,5 (13,7)	72,9 (13,2)	63,1 (10,2)	64,1 (13)
Zengin et al. 2019	N/A		67,7 (11,7)*	57,5 (19,6)*	N/A		N/A	
OP|NON operative/nonoperative; $^{\$}$ number (%); $^{\#}$ mean (SD); N/A not available; * percentage of unaffected side; ** difference between fractured side and unaffected side; ^ median								

©2020 Ochen Y et al. *JAMA Network Open*.

**eTable 7.** Clinical Outcome Measures of Included Studies in a Meta-analysis of Distal Radius Fractures								
**Study**	**Range wrist pronation (°)#**		**Range wrist supination (°)#**		**Radial deviation (°)#**		**Ulnar deviation (°)#**	
	**OP**	**NON**	**OP**	**NON**	**OP**	**NON**	**OP**	**NON**
**RCTs**								
Abbaszadegan et al. 1990	N/A		N/A		N/A		N/A	
Arora et al. 2011	84 (7)	85 (8)	85(8)	85 (8)	24 (6)	25 (7)	35 (8)	35 (8)
Azzopardi et al. 2005	100 (2)*	97 (6)*	91 (19)*	95 (7)*	89 (15)*	80 (31)*	93 (12)*	76 (26)*
Bartl et al. 2014	2,8 (5,6)**	2,6 (9,4)**	2,5 (5,9)**	3,2 (8,3)**	3,9 (6,3)**	3,0 (5,7)**	4,4 (7,5)**	5,9 (8,0)**
Martinez-Mendez et al. 2018	84 (10)	71 (19)	85 (5)	72 (20)	N/A		N/A	
Mulders et al. 2019	90 (7,4^)	85 (11,1)^	85 (11,1)^	75 (11,1)^	15 (7,4)^	15 (3,7)^	25 (4,4)^	25 (7,4)^
Sharma et al. 2014	34,1 (2,6)	32,0 (2,9)	43,4 (3,5)	41,9 (3,9)	79,1 (3,7)	62,8 (6,6)	79,6 (3,0)	65,9 (5,4)
Sirniö et al. 2019	88 (5)	88 (6)	88 (5)	84 (10)	22 (6)	22 (5)	28 (5)	25 (6)
**Observational studies**								
Aktekin et al. 2010	62	60	51	53	13	14	18	11
Alm-Paulsen et al. 2012	N/A		N/A		N/A		N/A	
Arora et al. 2009	82,2 (8,9)	81,4 (8,6)	83,0 (9,9)	82,5 (6,8)	20,6 (8,6)	21,2 (8,4)	38,0 (9,4)	36,4 (9,2)
Barai et al. 2018	N/A		N/A		N/A		N/A	
Chan et al. 2014	N/A		N/A		N/A		N/A	
Egol et al. 2010	82,9 (6,8)	84,4 (3,8)	80,6 (8,1)	83,9 (3,0)	18,7 (7,9)	22,9 (13,4)	29,9 (8,8)	30,3 (7,1)
Gong et al. 2011	N/A		N/A		N/A		N/A	
Hung et al. 2015	85^	90^	90^	80^	N/A		N/A	
Jordan et al. 2016	N/A		N/A		N/A		N/A	
Larouche et al. 2016	N/A		N/A		N/A		N/A	
Leerdam et al. 2019	N/A		N/A		N/A		N/A	
Lutz et al. 2014	N/A		N/A		N/A		N/A	
Tan et al. 2012	N/A		N/A		N/A		N/A	
Toon et al. 2017	N/A		N/A		15,6 (7,3)	15,7 (5,2)	22,8 (8,0)	17,9 (6,0)
Zengin et al. 2019	N/A		N/A		N/A		N/A	
OP|NON operative/nonoperative; $ number (%); # mean (SD); N/A not available; * percentage of unaffected side; ** difference between fractured side and unaffected side; ^ median								

©2020 Ochen Y et al. *JAMA Network Open*.

**eTable 8.** Radiologic Outcome Measures of Included Studies in a Meta-analysis of Distal Radius Fractures										
**Study**	**Volar tilt (°)#**		**Radial inclination (°)#**		**Radial height (mm)#**		**Articular step-off (mm)#**		**Ulnar variance (mm)#**	
	**OP**	**NON**	**OP**	**NON**	**OP**	**NON**	**OP**	**NON**	**OP**	**NON**
**RCTs**										
Abbaszadegan et al. 1990	N/A		N/A		N/A		N/A		N/A	
Arora et al. 2011	3,0 (7,2)	−10,4 (19,1)	21,2 (2,6)	15,9 (9,0)	N/A		0,2 (0,5)	0,6 (1,1)	0,7 (1,8)	3,2 (2,9)
Azzopardi et al. 2005	N/A		22,5 (5)	19 (6)	8 (3)	5 (4)	N/A		3 (2)	3 (2)
Bartl et al. 2014	N/A		20,3 (4,5)	17,7 (6,3)	N/A		N/A		N/A	
Martinez-Mendez et al. 2018	8 (8)	4 (8)	19 (6)	13 (6)	9 (2)	5 (3)	0,4 (1)	1 (3)	4 (3)	1 (2)
Mulders et al. 2019	N/A		N/A		N/A		N/A		N/A	
Sharma et al. 2014	8,3 (1,0)	5,1 (0,5)	17,8 (0,8)	15,2 (0,7)	8,2 (0,6)	6,1 (1,1)	N/A		−0,2 (0,2)	0,2 (0,0)
Sirniö et al. 2019	N/A		24 (4)	21 (8)	N/A		N/A		−0,7 (0,9)	−2,0 (1,7)
**Observational studies**										
Aktekin et al. 2010	11	8	18	20	10	8	N/A		0,27	0,5
Alm-Paulsen et al. 2012	N/A		21 (6)	19 (7)	7 (4)	6 (5)	N/A		3 (2)	3 (3)
Arora et al. 2009	N/A		23,6 (3,8)	19,2 (6,9)	N/A		N/A		1,5 (1,9)	3,8 (2,6)
Barai et al. 2018	N/A		N/A		N/A		N/A		N/A	
Chan et al. 2014	N/A		N/A		N/A		N/A		N/A	
Egol et al. 2010	6,2 (9,2)	−5,8 (10,4)	22,3 (4,7)	18,0 (4,0)	10,6 (2,5)	8,7 (1,6)	N/A		1,5 (2,2)	2,8 (1,8)
Gong et al. 2011	8,1 (1,5)	5,3 (3,4)	19,2 (2,2)	14,3 (5,6)	9,5 (1,3)	6,3 (3,2)	N/A		N/A	
Hung et al. 2015	9,6 (7,3)	−5,2 (15)	21,0 (4,6)	17,9 (5,0)	9,8 (2,4)	8,6 (2,6)	N/A		N/A	
Jordan et al. 2016	N/A		N/A		10,9 (2,6)	8,2 (2,2)	0,9 (0,5)	0,9 (0,6)	N/A	
Larouche et al. 2016	8,3 (7,9)	1,3 (12,3)	24,2 (5,2)	21,2 (6,0)	11,1 (3,4)	9,7 (3,4)	0,1 (0,4)	0,1 (0,4)	−0,8 (2,4)	−1,6 (2,8)
Leerdam et al. 2019	N/A		N/A		N/A		N/A		N/A	
Lutz et al. 2014	2 (9)	7 (13)	20 (5)	19 (6)	N/A		0,3 (0,7)	0,2 (0,6)	1,3 (2,1)	2,6 (2,2)
Tan et al. 2012	N/A		N/A		N/A		N/A		N/A	
Toon et al. 2017	5,6 (8,9)	0,1 (11,6)	21,6 (6,1)	16,9 (6,3)	9,6 (3,3)	7,2 (3,4)	0,7 (0,6)	1,5 (0,9)	N/A	
Zengin et al. 2019	N/A		21,5 (2,6)	16,6 (5,3)	10,4 (2,8)	7,8 (2,4)	0,6 (0,5)	1,4 (0,9)	1,7 (1,7)	2,1 (2,1)
OP|NON operative/nonoperative; n number; # mean (SD); N/A not available										

©2020 Ochen Y et al. *JAMA Network Open*.

eFigure 1. PRISMA Flow Diagram Representing the Search and Selection of Studies Comparing Operative vs Nonoperative Treatment of Distal Radius Fractures

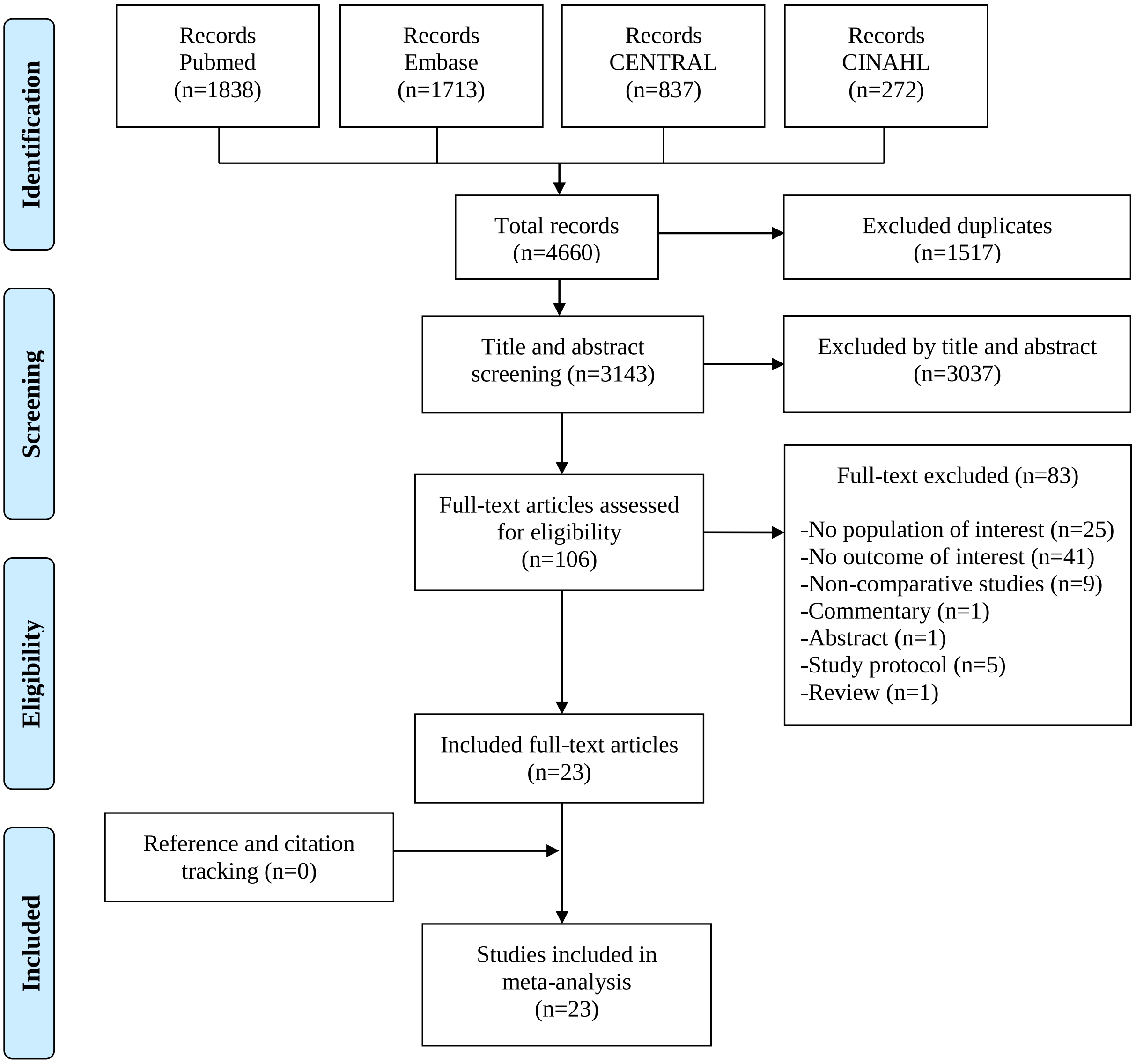

©2020 Ochen Y et al. *JAMA Network Open*.

eFigure 2. Funnel Plot of Medium-Term (≤ 1 y) DASH Score in a Meta-analysis of Distal Radius Fractures (MD mean difference; SE standard error).

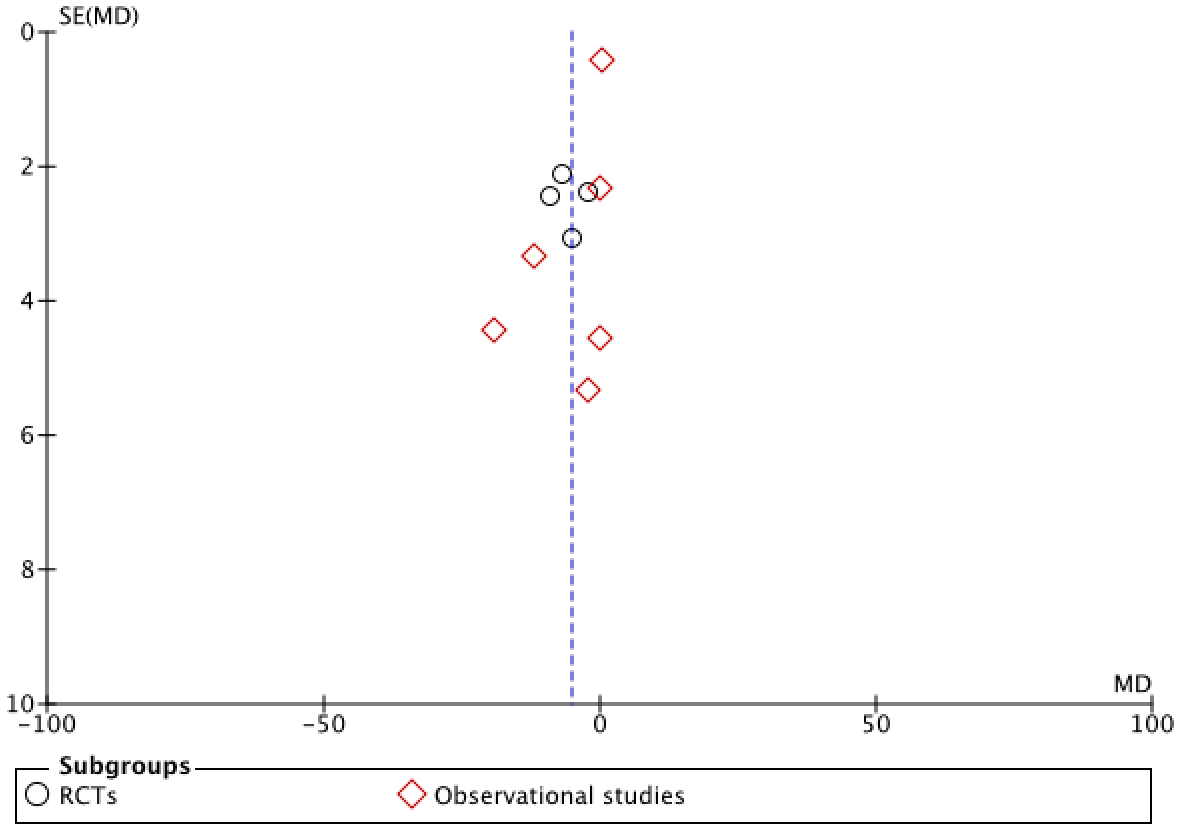

©2020 Ochen Y et al. *JAMA Network Open*.

eFigure 3. Funnel Plot of Complication Rate in a Meta-analysis of Distal Radius Fractures

(RR risk ratio; SE standard error).

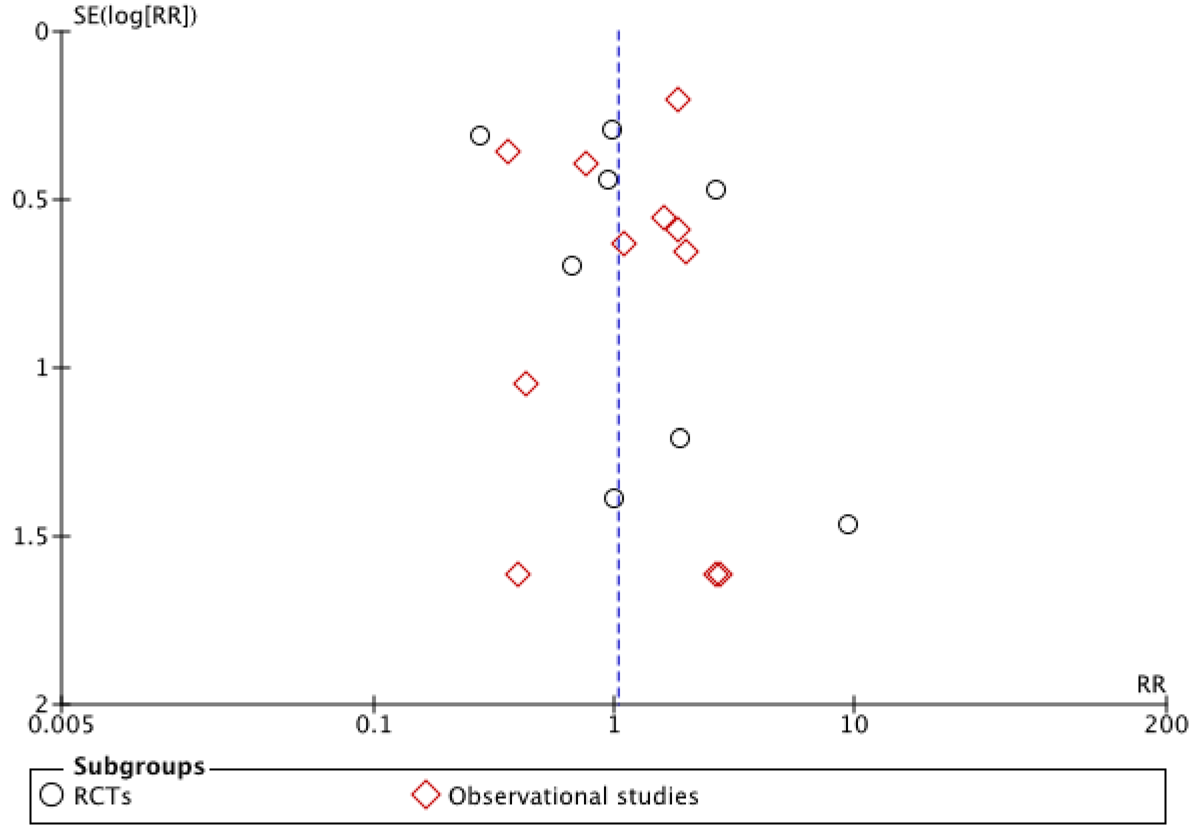

©2020 Ochen Y et al. *JAMA Network Open*.

eFigure 4. Forest Plot of Long-Term (> 1 y) DASH Score in a Meta-analysis of Distal Radius Fractures

Study or Subgroup	Operative Mean	Operative SD	Operative Total	Nonoperative Mean	Nonoperative SD	Nonoperative Total	Weight	Mean Difference IV, Random, 95% CI
**1.4.1 RCTs**								
Martinez–Mendez et al.2018	16	14	50	28	21	47	11.9%	−12.00 [−19.15, −4.85]
**Subtotal (95% CI)**			**50**			**47**	**11.9%**	**−12.00 [−19.15, −4.85]**
Heterogeneity: Not applicable								
Test for overall effect: Z = 3.29 (P = 0.001)								
**1.4.2 Observational studies**								
Aktekin et al.2010	21.9	21.7	22	20.3	13	24	8.8%	1.60 [−8.85, 12.05]
Alm–Paulsen et al.2012	13.5	15	30	10	13	30	12.0%	3.50 [−3.60, 10.60]
Arora et al.2009	11.1	12.9	53	11.6	13.4	61	14.4%	−0.50 [−5.33, 4.33]
Barai et al.2018	12.1	14.4	29	6	10.9	87	13.5%	6.10 [0.38, 11.82]
Jordan et al.2016	26.6	6.5	74	27.1	7.7	85	16.7%	−0.50 [−2.71, 1.71]
Tan et al.2012	7	9	31	25	24	32	10.2%	−18.00 [−26.90, −9.10]
Zengin et al.2019	11.7	8	25	17.6	14.2	24	12.6%	−5.90 [−12.39, 0.59]
**Subtotal (95% CI)**			**264**			**343**	**88.1%**	**−1.45 [−5.72, 2.81]**
Heterogeneity: $Tau^2 = 22.29$; $Chi^2 = 23.98$, df = 6 (P = 0.0005); $I^2 = 75\%$								
Test for overall effect: Z = 0.67 (P = 0.50)								
**Total (95% CI)**			**314**			**390**	**100.0%**	**−2.78 [−7.18, 1.62]**
Heterogeneity: $Tau^2 = 28.89$; $Chi^2 = 33.15$, df = 7 (P < 0.0001); $I^2 = 79\%$								
Test for overall effect: Z = 1.24 (P = 0.22)								
Test for subgroup differences: $Chi^2 = 6.17$, df = 1 (P = 0.01), $I^2 = 83.8\%$								

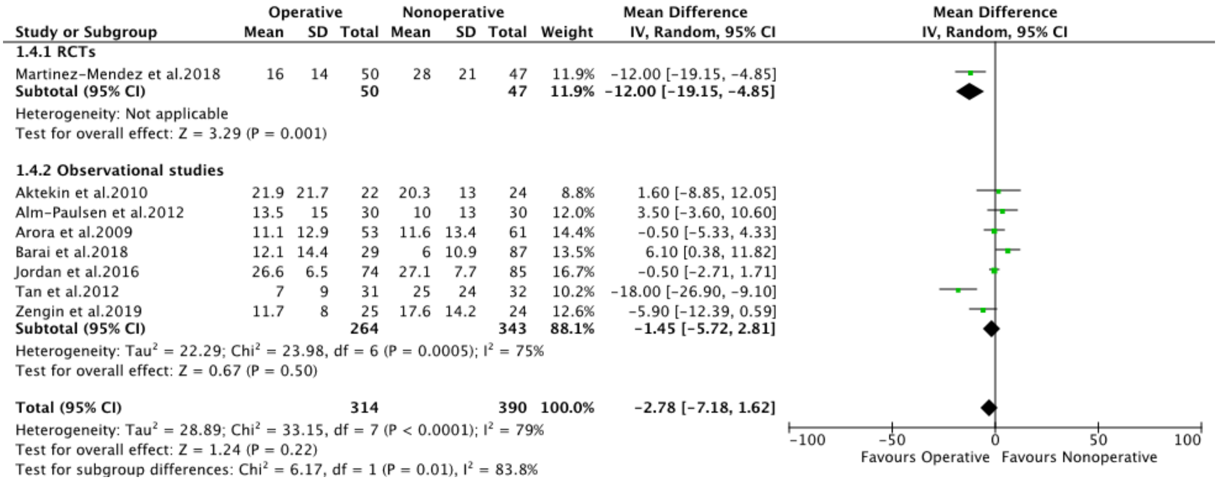

©2020 Ochen Y et al. *JAMA Network Open*.

eFigure 5. Forest Plot of Medium-Term (≤ 1 y) PRWE Score in a Meta-analysis of Distal Radius Fractures

Study or Subgroup	Operative Mean	Operative SD	Operative Total	Nonoperative Mean	Nonoperative SD	Nonoperative Total	Weight	Mean Difference IV, Random, 95% CI
**1.5.1 RCTs**								
Arora et al.2011	12.8	23.2	36	14.6	22.8	37	11.3%	−1.80 [−12.35, 8.75]
Mulders et al.2019	4	9.3	48	8	11.9	44	49.6%	−4.00 [−8.39, 0.39]
**Subtotal (95% CI)**			**84**			**81**	**60.9%**	**−3.68 [−7.73, 0.38]**
Heterogeneity: $Tau^2 = 0.00$; $Chi^2 = 0.14$, df = 1 (P = 0.71); $I^2 = 0\%$								
Test for overall effect: Z = 1.78 (P = 0.08)								
**1.5.2 Observational studies**								
Larouche et al.2016	12.3	15.8	70	10.9	14.1	59	39.1%	1.40 [−3.76, 6.56]
**Subtotal (95% CI)**			**70**			**59**	**39.1%**	**1.40 [−3.76, 6.56]**
Heterogeneity: Not applicable								
Test for overall effect: Z = 0.53 (P = 0.60)								
**Total (95% CI)**			**154**			**140**	**100.0%**	**−1.64 [−5.31, 2.03]**
Heterogeneity: $Tau^2 = 2.05$; $Chi^2 = 2.44$, df = 2 (P = 0.30); $I^2 = 18\%$								
Test for overall effect: Z = 0.88 (P = 0.38)								
Test for subgroup differences: $Chi^2 = 2.30$, df = 1 (P = 0.13), $I^2 = 56.5\%$								

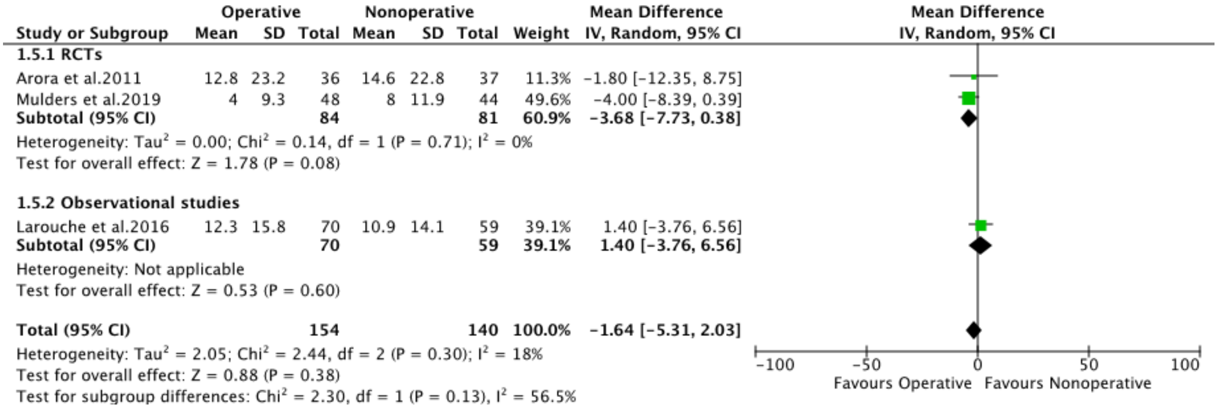

©2020 Ochen Y et al. *JAMA Network Open*.

eFigure 6. Forest Plot of Long-Term (>1 y) PRWE Score in a Meta-analysis of Distal Radius Fractures

Study or Subgroup	Operative Mean	Operative SD	Operative Total	Nonoperative Mean	Nonoperative SD	Nonoperative Total	Weight	Mean Difference IV, Random, 95% CI
**1.1.1 RCTs**								
Martinez–Mendez et al.2018	17	13	50	30	25	47	18.0%	−13.00 [−21.00, −5.00]
**Subtotal (95% CI)**			**50**			**47**	**18.0%**	**−13.00 [−21.00, −5.00]**
Heterogeneity: Not applicable								
Test for overall effect: Z = 3.18 (P = 0.001)								
**1.1.2 Observational studies**								
Alm–Paulsen et al.2012	11	15	30	8	12	30	19.1%	3.00 [−3.87, 9.87]
Arora et al.2009	9.3	9.3	53	16.9	12.1	61	21.5%	−7.60 [−11.54, −3.66]
Leerdam et al.2019	17	22	87	8	15	185	20.6%	9.00 [3.90, 14.10]
Lutz et al.2014	17	23	129	16	18	129	20.7%	1.00 [−4.04, 6.04]
**Subtotal (95% CI)**			**299**			**405**	**82.0%**	**1.21 [−6.45, 8.86]**
Heterogeneity: $Tau^2 = 53.76$; $Chi^2 = 27.11$, df = 3 (P < 0.00001); $I^2 = 89\%$								
Test for overall effect: Z = 0.31 (P = 0.76)								
**Total (95% CI)**			**349**			**452**	**100.0%**	**−1.34 [−8.71, 6.03]**
Heterogeneity: $Tau^2 = 61.67$; $Chi^2 = 36.00$, df = 4 (P < 0.00001); $I^2 = 89\%$								
Test for overall effect: Z = 0.36 (P = 0.72)								
Test for subgroup differences: $Chi^2 = 6.32$, df = 1 (P = 0.01), $I^2 = 84.2\%$								

Mean Difference IV, Random, 95% CI

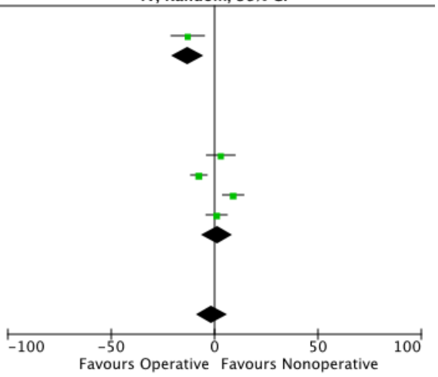

©2020 Ochen Y et al. *JAMA Network Open*.

eFigure 7. Forest Plot of Medium-Term (≤1 y) VAS Score in a Meta-analysis of Distal Radius Fractures

	Operative			Nonoperative				Mean Difference
Study or Subgroup	Mean	SD	Total	Mean	SD	Total	Weight	IV, Random, 95% CI
**1.6.1 RCTs**								
Arora et al.2011	0.1	0.3	36	0.1	0.5	37	45.0%	0.00 [−0.19, 0.19]
Azzopardi et al.2005	0.7	1.3	27	1.2	1.6	27	17.5%	−0.50 [−1.28, 0.28]
**Subtotal (95% CI)**			**63**			**64**	**62.5%**	**−0.10 [−0.50, 0.29]**
Heterogeneity: $Tau^2 = 0.04$; $Chi^2 = 1.50$, df = 1 (P = 0.22); $I^2 = 33\%$								
Test for overall effect: Z = 0.51 (P = 0.61)								
**1.6.2 Observational studies**								
Egol et al.2010	1.2	1.7	44	1.5	2.1	46	17.2%	−0.30 [−1.09, 0.49]
Toon et al.2017	1.8	1.6	32	1.1	1.1	28	20.3%	0.70 [0.01, 1.39]
**Subtotal (95% CI)**			**76**			**74**	**37.5%**	**0.22 [−0.76, 1.20]**
Heterogeneity: $Tau^2 = 0.36$; $Chi^2 = 3.51$, df = 1 (P = 0.06); $I^2 = 72\%$								
Test for overall effect: Z = 0.44 (P = 0.66)								
**Total (95% CI)**			**139**			**138**	**100.0%**	**0.00 [−0.40, 0.41]**
Heterogeneity: $Tau^2 = 0.08$; $Chi^2 = 6.12$, df = 3 (P = 0.11); $I^2 = 51\%$								
Test for overall effect: Z = 0.02 (P = 0.99)								
Test for subgroup differences: $Chi^2 = 0.36$, df = 1 (P = 0.55), $I^2 = 0\%$								

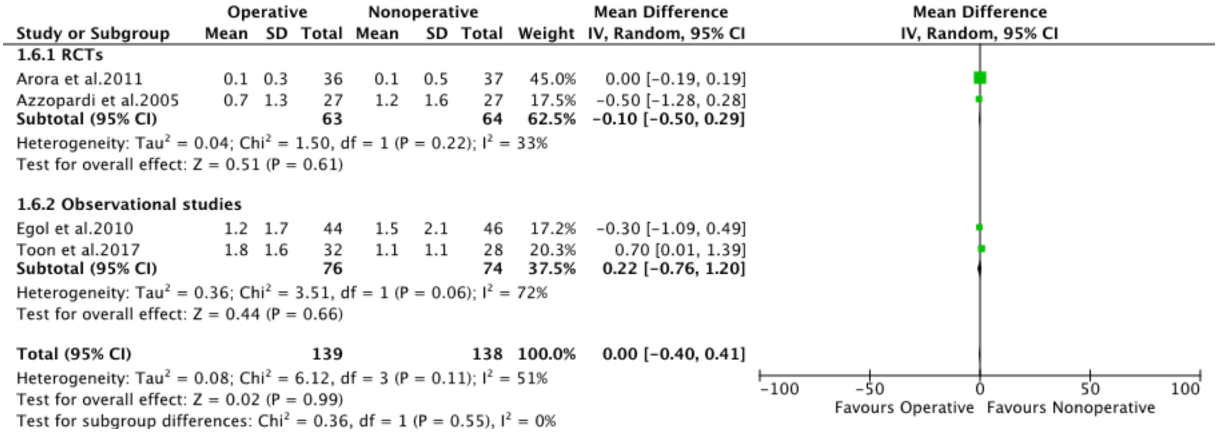

©2020 Ochen Y et al. *JAMA Network Open*.

eFigure 8. Forest Plot of Long-Term (>1 y) VAS Score in a Meta-analysis of Distal Radius Fractures

	Operative			Nonoperative				Mean Difference
**Study or Subgroup**	**Mean**	**SD**	**Total**	**Mean**	**SD**	**Total**	**Weight**	**IV, Random, 95% CI**
**1.7.1 RCTs**								
Martinez–Mendez et al.2018	2	2	50	3	2	47	43.1%	−1.00 [−1.80, −0.20]
**Subtotal (95% CI)**			**50**			**47**	**43.1%**	**−1.00 [−1.80, −0.20]**
Heterogeneity: Not applicable								
Test for overall effect: Z = 2.46 (P = 0.01)								
**1.7.2 Observational studies**								
Alm–Paulsen et al.2012	5	11	30	2	7	30	11.5%	3.00 [−1.67, 7.67]
Arora et al.2009	1.7	1.4	53	0.7	1.4	61	45.3%	1.00 [0.48, 1.52]
**Subtotal (95% CI)**			**83**			**91**	**56.9%**	**1.02 [0.51, 1.54]**
Heterogeneity: $Tau^2 = 0.00$; $Chi^2 = 0.70$, df = 1 (P = 0.40); $I^2 = 0\%$								
Test for overall effect: Z = 3.92 (P < 0.0001)								
**Total (95% CI)**			**133**			**138**	**100.0%**	**0.37 [−1.46, 2.19]**
Heterogeneity: $Tau^2 = 1.84$; $Chi^2 = 18.25$, df = 2 (P = 0.0001); $I^2 = 89\%$								
Test for overall effect: Z = 0.40 (P = 0.69)								
Test for subgroup differences: $Chi^2 = 17.55$, df = 1 (P < 0.0001), $I^2 = 94.3\%$								

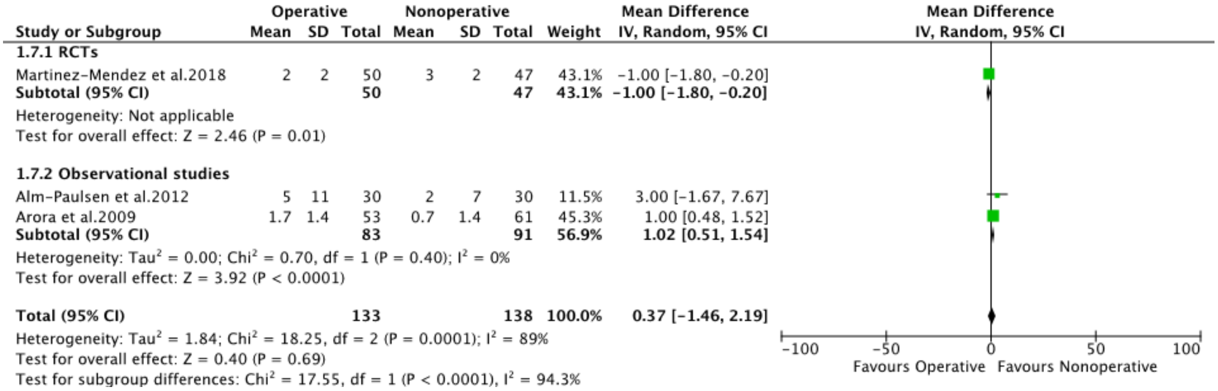

©2020 Ochen Y et al. *JAMA Network Open*.

eFigure 9. Forest Plot of Grip Strength (in kg) in a Meta-analysis of Distal Radius Fractures

	Operative			Nonoperative				Mean Difference
Study or Subgroup	Mean	SD	Total	Mean	SD	Total	Weight	IV, Random, 95% CI
**1.8.1 RCTs**								
Arora et al.2011	22.2	6.3	36	18.8	5.8	37	17.5%	3.40 [0.62, 6.18]
Mulders et al.2019	26	1.9	48	20	8.4	44	18.1%	6.00 [3.46, 8.54]
Sirniö et al.2019	27	5	38	26	7	42	17.8%	1.00 [−1.65, 3.65]
**Subtotal (95% CI)**			**122**			**123**	**53.4%**	**3.49 [0.59, 6.38]**
Heterogeneity: $Tau^2 = 4.72$; $Chi^2 = 7.15$, df = 2 (P = 0.03); $I^2 = 72\%$								
Test for overall effect: Z = 2.36 (P = 0.02)								
**1.8.2 Observational studies**								
Alm-Paulsen et al.2012	30	12	30	27	10	30	10.8%	3.00 [−2.59, 8.59]
Arora et al.2009	19.4	6	53	21.1	7	61	18.5%	−1.70 [−4.09, 0.69]
Egol et al.2010	17.7	7.3	44	12.7	6.5	46	17.3%	5.00 [2.14, 7.86]
**Subtotal (95% CI)**			**127**			**137**	**46.6%**	**1.96 [−2.91, 6.83]**
Heterogeneity: $Tau^2 = 15.03$; $Chi^2 = 12.85$, df = 2 (P = 0.002); $I^2 = 84\%$								
Test for overall effect: Z = 0.79 (P = 0.43)								
**Total (95% CI)**			**249**			**260**	**100.0%**	**2.73 [0.15, 5.32]**
Heterogeneity: $Tau^2 = 7.96$; $Chi^2 = 23.79$, df = 5 (P = 0.0002); $I^2 = 79\%$								
Test for overall effect: Z = 2.07 (P = 0.04)								
Test for subgroup differences: $Chi^2 = 0.28$, df = 1 (P = 0.60), $I^2 = 0\%$								

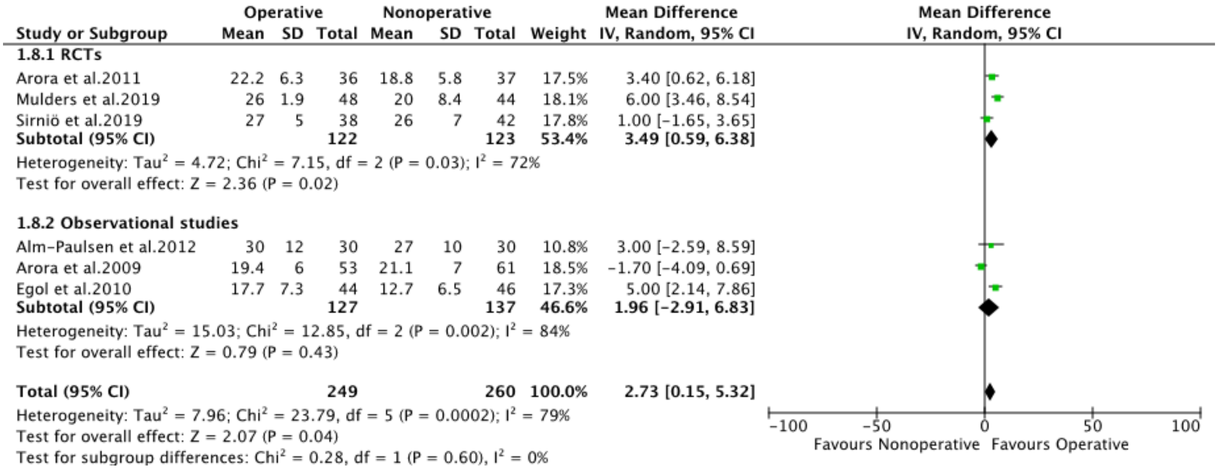

©2020 Ochen Y et al. *JAMA Network Open*.

eFigure 10. Forest Plot of Grip Strength as Percentage of Unaffected Side in a Meta-analysis of Distal Radius Fractures

Study or Subgroup	Operative Mean	Operative SD	Operative Total	Nonoperative Mean	Nonoperative SD	Nonoperative Total	Weight	Mean Difference IV, Random, 95% CI
**1.20.1 RCTs**								
Azzopardi et al.2005	77	21	27	72	17	27	13.0%	5.00 [−5.19, 15.19]
Martinez−Mendez et al.2018	73	27	50	64	33	47	11.3%	9.00 [−3.04, 21.04]
Sharma et al.2014	89	4.3	32	72.1	4.4	32	20.4%	16.90 [14.77, 19.03]
**Subtotal (95% CI)**			**109**			**106**	**44.6%**	**11.62 [3.29, 19.95]**
Heterogeneity: Tau² = 36.82; Chi² = 6.43, df = 2 (P = 0.04); I² = 69%								
Test for overall effect: Z = 2.74 (P = 0.006)								
**1.20.2 Observational studies**								
Chan et al.2014	82	19	40	77	17	35	15.0%	5.00 [−3.15, 13.15]
Tan et al.2012	83	17	31	78	24	32	12.9%	5.00 [−5.24, 15.24]
Toon et al.2017	83.3	14.1	32	81.3	22.9	28	13.4%	2.00 [−7.79, 11.79]
Zengin et al.2019	67.7	11.7	25	57.5	19.6	24	14.1%	10.20 [1.12, 19.28]
**Subtotal (95% CI)**			**128**			**119**	**55.4%**	**5.67 [1.07, 10.28]**
Heterogeneity: Tau² = 0.00; Chi² = 1.54, df = 3 (P = 0.67); I² = 0%								
Test for overall effect: Z = 2.41 (P = 0.02)								
**Total (95% CI)**			**237**			**225**	**100.0%**	**8.21 [2.26, 14.15]**
Heterogeneity: Tau² = 43.93; Chi² = 24.66, df = 6 (P = 0.0004); I² = 76%								
Test for overall effect: Z = 2.71 (P = 0.007)								
Test for subgroup differences: Chi² = 1.50, df = 1 (P = 0.22), I² = 33.4%								

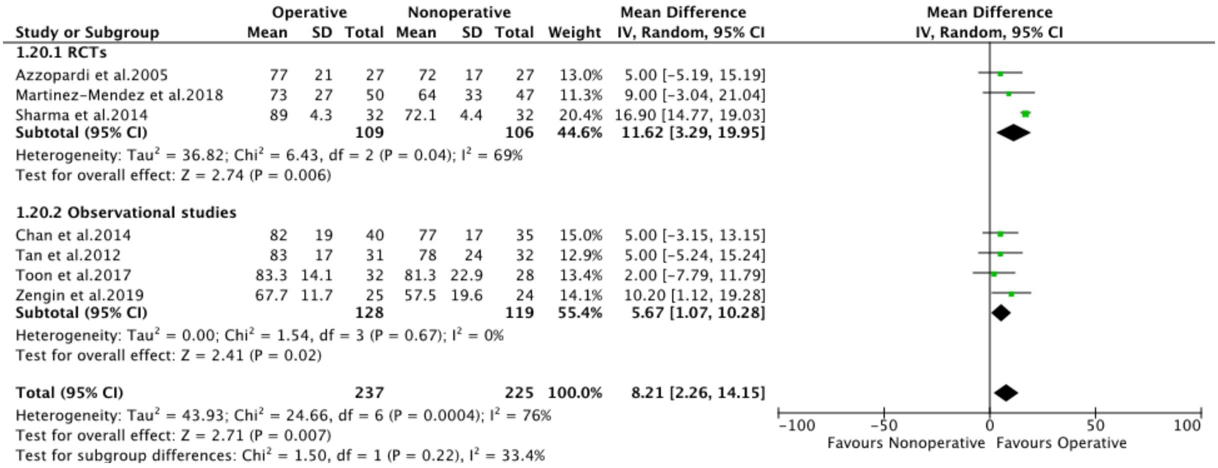

©2020 Ochen Y et al. *JAMA Network Open*.

eFigure 11. Forest Plot of Range of Wrist Extension in Degrees in a Meta-analysis of Distal Radius Fractures

Study or Subgroup	Operative Mean	Operative SD	Operative Total	Nonoperative Mean	Nonoperative SD	Nonoperative Total	Weight	Mean Difference IV, Random, 95% CI
**1.9.1 RCTs**								
Arora et al.2011	59	10	36	61	7	37	12.7%	−2.00 [−5.97, 1.97]
Martinez−Mendez et al.2018	57	11	50	54	13	47	12.5%	3.00 [−1.81, 7.81]
Mulders et al.2019	85	7.4	48	80	14.8	44	12.4%	5.00 [0.15, 9.85]
Sharma et al.2014	84.3	2.4	32	69	3.91	32	13.2%	15.30 [13.71, 16.89]
Sirniö et al.2019	69	6	38	68	7	42	13.0%	1.00 [−1.85, 3.85]
**Subtotal (95% CI)**			**204**			**202**	**63.9%**	**4.54 [−3.52, 12.59]**
Heterogeneity: $Tau^2 = 80.71$; $Chi^2 = 127.88$, df = 4 (P < 0.00001); $I^2 = 97\%$								
Test for overall effect: Z = 1.10 (P = 0.27)								
**1.9.2 Observational studies**								
Arora et al.2009	57	11.6	53	59.8	7	61	12.8%	−2.80 [−6.38, 0.78]
Egol et al.2010	54.8	18.7	44	54.6	14.9	46	11.6%	0.20 [−6.81, 7.21]
Toon et al.2017	67.5	13.7	32	72.9	13.2	28	11.7%	−5.40 [−12.21, 1.41]
**Subtotal (95% CI)**			**129**			**135**	**36.1%**	**−2.76 [−5.65, 0.13]**
Heterogeneity: $Tau^2 = 0.00$; $Chi^2 = 1.26$, df = 2 (P = 0.53); $I^2 = 0\%$								
Test for overall effect: Z = 1.87 (P = 0.06)								
**Total (95% CI)**			**333**			**337**	**100.0%**	**1.93 [−4.69, 8.55]**
Heterogeneity: $Tau^2 = 85.66$; $Chi^2 = 188.44$, df = 7 (P < 0.00001); $I^2 = 96\%$								
Test for overall effect: Z = 0.57 (P = 0.57)								
Test for subgroup differences: $Chi^2 = 2.79$, df = 1 (P = 0.09), $I^2 = 64.2\%$								

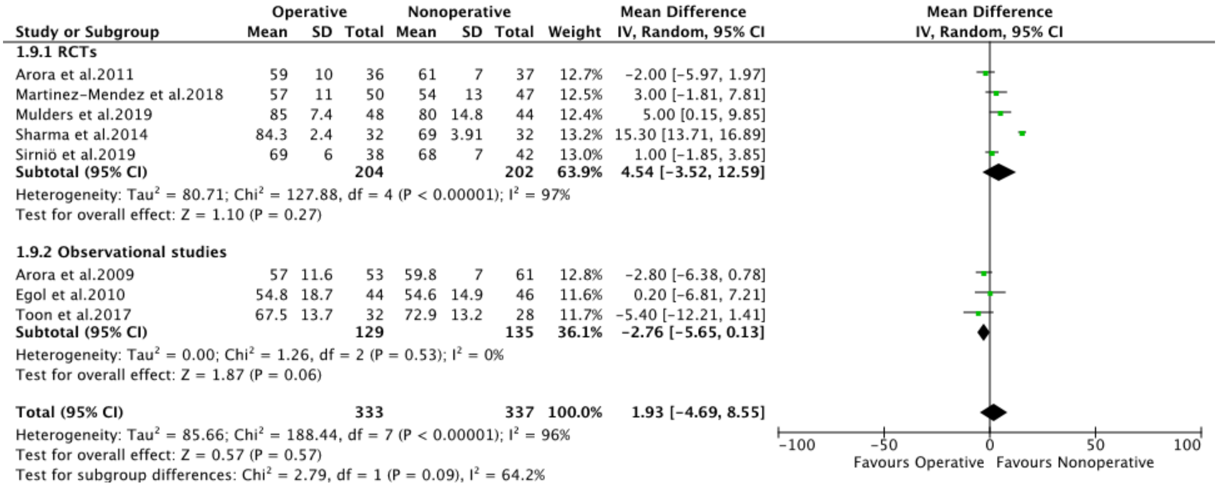

©2020 Ochen Y et al. *JAMA Network Open*.

eFigure 12. Forest Plot of Range of Wrist Flexion in Degrees in a Meta-analysis of Distal Radius Fractures

Study or Subgroup	Operative Mean	Operative SD	Operative Total	Nonoperative Mean	Nonoperative SD	Nonoperative Total	Weight	Mean Difference IV, Random, 95% CI
**1.10.1 RCTs**								
Arora et al.2011	55	11	36	57	10	37	12.5%	−2.00 [−6.83, 2.83]
Martinez–Mendez et al.2018	54	13	50	60	16	47	12.2%	−6.00 [−11.82, −0.18]
Mulders et al.2019	80	11.9	48	70	14.8	44	12.3%	10.00 [4.48, 15.52]
Sharma et al.2014	83.8	2.9	32	65.9	7.5	32	13.0%	17.90 [15.11, 20.69]
Sirniö et al.2019	71	7	38	64	11	42	12.7%	7.00 [3.00, 11.00]
**Subtotal (95% CI)**			**204**			**202**	**62.6%**	**5.53 [−3.44, 14.49]**
Heterogeneity: $Tau^2 = 98.83$; $Chi^2 = 85.48$, df = 4 (P < 0.00001); $I^2 = 95\%$								
Test for overall effect: Z = 1.21 (P = 0.23)								
**1.10.2 Observational studies**								
Arora et al.2009	44.6	10.4	53	49.6	9.8	61	12.8%	−5.00 [−8.73, −1.27]
Egol et al.2010	47.8	13.1	44	51.8	11.1	46	12.4%	−4.00 [−9.03, 1.03]
Toon et al.2017	63.1	10.2	32	64.1	13	28	12.1%	−1.00 [−6.97, 4.97]
**Subtotal (95% CI)**			**129**			**135**	**37.4%**	**−3.91 [−6.59, −1.24]**
Heterogeneity: $Tau^2 = 0.00$; $Chi^2 = 1.24$, df = 2 (P = 0.54); $I^2 = 0\%$								
Test for overall effect: Z = 2.87 (P = 0.004)								
**Total (95% CI)**			**333**			**337**	**100.0%**	**2.20 [−5.07, 9.47]**
Heterogeneity: $Tau^2 = 103.98$; $Chi^2 = 152.05$, df = 7 (P < 0.00001); $I^2 = 95\%$								
Test for overall effect: Z = 0.59 (P = 0.55)								
Test for subgroup differences: $Chi^2 = 3.91$, df = 1 (P = 0.05), $I^2 = 74.4\%$								

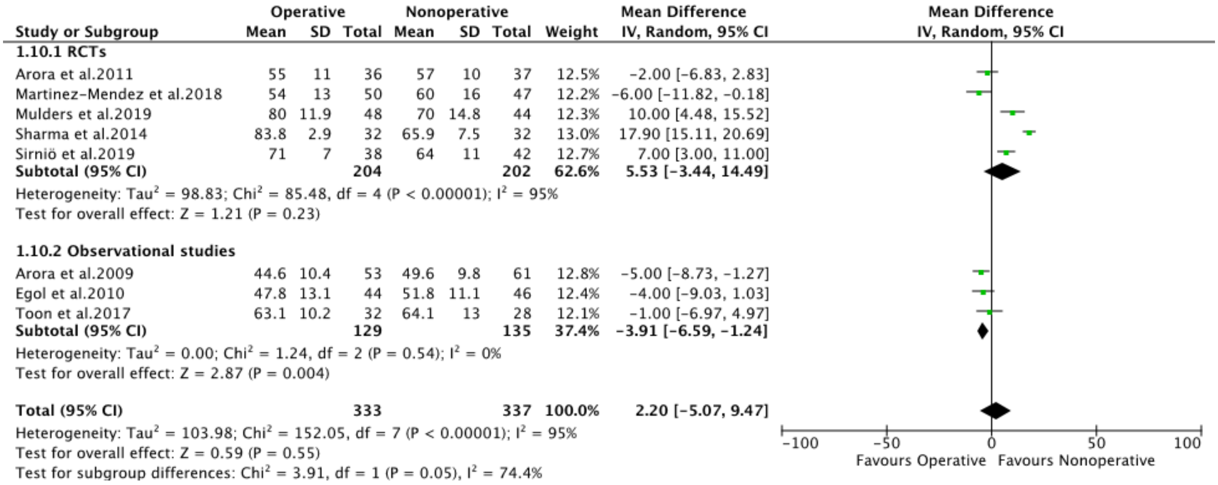

©2020 Ochen Y et al. *JAMA Network Open*.

eFigure 13. Forest Plot of Range of Wrist Pronation in Degrees in a Meta-analysis of Distal Radius Fractures

Study or Subgroup	Operative Mean	Operative SD	Operative Total	Nonoperative Mean	Nonoperative SD	Nonoperative Total	Weight	Mean Difference IV, Random, 95% CI
**1.11.1 RCTs**								
Arora et al.2011	84	7	36	85	8	37	13.6%	−1.00 [−4.45, 2.45]
Martinez−Mendez et al.2018	84	10	50	71	19	47	8.1%	13.00 [6.90, 19.10]
Mulders et al.2019	90	7.4	48	85	11.1	44	12.5%	5.00 [1.11, 8.89]
Sharma et al.2014	34.1	2.6	32	32	2.9	32	18.7%	2.10 [0.75, 3.45]
Sirniö et al.2019	88	5	38	88	6	42	16.3%	0.00 [−2.41, 2.41]
**Subtotal (95% CI)**			**204**			**202**	**69.2%**	**2.91 [−0.04, 5.85]**
Heterogeneity: $Tau^2 = 8.25$; $Chi^2 = 20.35$, df = 4 (P = 0.0004); $I^2 = 80\%$								
Test for overall effect: Z = 1.93 (P = 0.05)								
**1.11.2 Observational studies**								
Arora et al.2009	82.2	8.9	53	81.4	8.6	61	14.2%	0.80 [−2.42, 4.02]
Egol et al.2010	82.9	6.8	44	84.4	3.8	46	16.6%	−1.50 [−3.79, 0.79]
**Subtotal (95% CI)**			**97**			**107**	**30.8%**	**−0.64 [−2.82, 1.54]**
Heterogeneity: $Tau^2 = 0.61$; $Chi^2 = 1.30$, df = 1 (P = 0.25); $I^2 = 23\%$								
Test for overall effect: Z = 0.58 (P = 0.56)								
**Total (95% CI)**			**301**			**309**	**100.0%**	**1.80 [−0.45, 4.05]**
Heterogeneity: $Tau^2 = 6.57$; $Chi^2 = 27.63$, df = 6 (P = 0.0001); $I^2 = 78\%$								
Test for overall effect: Z = 1.57 (P = 0.12)								
Test for subgroup differences: $Chi^2 = 3.60$, df = 1 (P = 0.06), $I^2 = 72.2\%$								

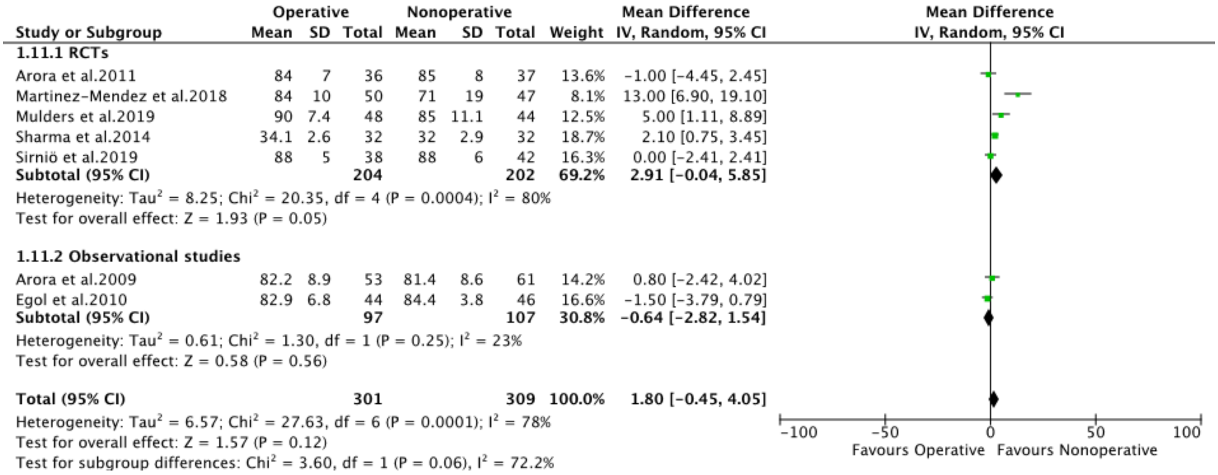

©2020 Ochen Y et al. *JAMA Network Open*.

eFigure 14. Forest Plot of Range of Wrist Supination in Degrees in a Meta-analysis of Distal Radius Fractures

	Operative			Nonoperative				Mean Difference
Study or Subgroup	Mean	SD	Total	Mean	SD	Total	Weight	IV, Random, 95% CI
**1.12.1 RCTs**								
Arora et al.2011	85	8	36	85	8	37	14.2%	0.00 [−3.67, 3.67]
Martinez–Mendez et al.2018	85	5	50	72	20	47	11.2%	13.00 [7.12, 18.88]
Mulders et al.2019	85	11.1	48	75	11.1	44	13.0%	10.00 [5.46, 14.54]
Sharma et al.2014	43.4	3.5	32	41.9	3.9	32	16.4%	1.50 [−0.32, 3.32]
Sirniö et al.2019	88	5	38	84	10	42	14.6%	4.00 [0.58, 7.42]
**Subtotal (95% CI)**			**204**			**202**	**69.4%**	**5.16 [1.18, 9.15]**
Heterogeneity: $Tau^2 = 16.60$; $Chi^2 = 25.74$, df = 4 (P < 0.0001); $I^2 = 84\%$								
Test for overall effect: Z = 2.54 (P = 0.01)								
**1.12.2 Observational studies**								
Arora et al.2009	83	9.9	53	82.5	6.8	61	14.9%	0.50 [−2.66, 3.66]
Egol et al.2010	80.6	8.1	44	83.9	3	46	15.7%	−3.30 [−5.85, −0.75]
**Subtotal (95% CI)**			**97**			**107**	**30.6%**	**−1.52 [−5.24, 2.20]**
Heterogeneity: $Tau^2 = 5.07$; $Chi^2 = 3.36$, df = 1 (P = 0.07); $I^2 = 70\%$								
Test for overall effect: Z = 0.80 (P = 0.42)								
**Total (95% CI)**			**301**			**309**	**100.0%**	**3.14 [−0.16, 6.44]**
Heterogeneity: $Tau^2 = 16.42$; $Chi^2 = 44.88$, df = 6 (P < 0.00001); $I^2 = 87\%$								
Test for overall effect: Z = 1.86 (P = 0.06)								
Test for subgroup differences: $Chi^2 = 5.79$, df = 1 (P = 0.02), $I^2 = 82.7\%$								

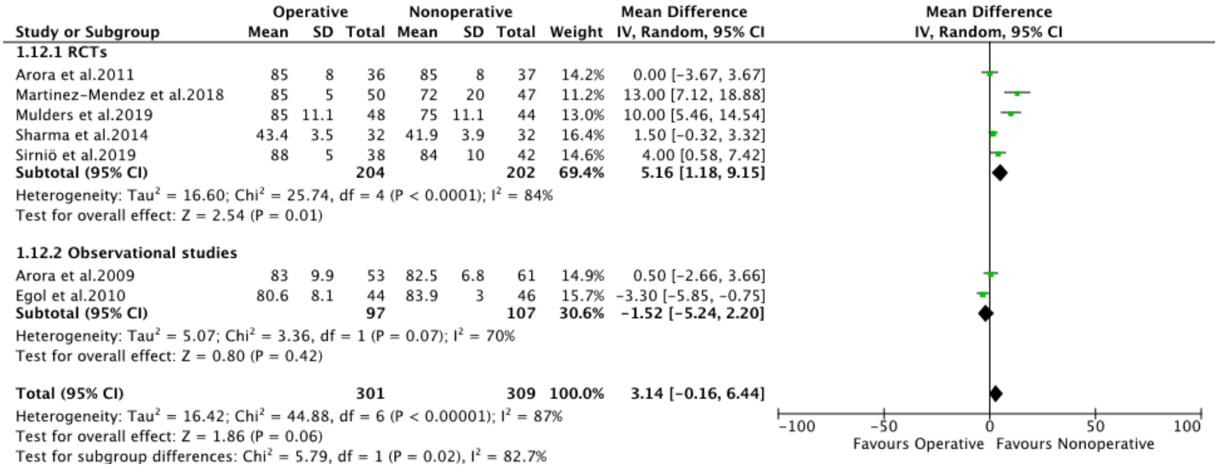

©2020 Ochen Y et al. *JAMA Network Open*.

eFigure 15. Forest Plot of Radial Deviation in Degrees in a Meta-analysis of Distal Radius Fractures

	Operative			Nonoperative				Mean Difference
**Study or Subgroup**	**Mean**	**SD**	**Total**	**Mean**	**SD**	**Total**	**Weight**	**IV, Random, 95% CI**
**1.13.1 RCTs**								
Arora et al.2011	24	6	36	25	7	37	14.3%	−1.00 [−3.99, 1.99]
Mulders et al.2019	15	7.4	48	15	3.7	44	14.6%	0.00 [−2.36, 2.36]
Sharma et al.2014	79	3.7	32	62.8	6.6	32	14.5%	16.20 [13.58, 18.82]
Sirniö et al.2019	22	6	38	22	5	42	14.6%	0.00 [−2.43, 2.43]
**Subtotal (95% CI)**			**154**			**155**	**58.0%**	**3.80 [−4.20, 11.80]**
Heterogeneity: $Tau^2 = 64.91$; $Chi^2 = 115.14$, df = 3 (P < 0.00001); $I^2 = 97\%$								
Test for overall effect: Z = 0.93 (P = 0.35)								
**1.13.2 Observational studies**								
Arora et al.2009	20.6	8.6	53	21.2	8.4	61	14.3%	−0.60 [−3.73, 2.53]
Egol et al.2010	18.7	7.9	44	22.9	13.4	46	13.5%	−4.20 [−8.72, 0.32]
Toon et al.2017	15.6	7.3	32	15.7	5.2	28	14.2%	−0.10 [−3.28, 3.08]
**Subtotal (95% CI)**			**129**			**135**	**42.0%**	**−1.16 [−3.31, 1.00]**
Heterogeneity: $Tau^2 = 0.46$; $Chi^2 = 2.28$, df = 2 (P = 0.32); $I^2 = 12\%$								
Test for overall effect: Z = 1.05 (P = 0.29)								
**Total (95% CI)**			**283**			**290**	**100.0%**	**1.54 [−3.62, 6.70]**
Heterogeneity: $Tau^2 = 46.06$; $Chi^2 = 133.18$, df = 6 (P < 0.00001); $I^2 = 95\%$								
Test for overall effect: Z = 0.58 (P = 0.56)								
Test for subgroup differences: $Chi^2 = 1.37$, df = 1 (P = 0.24), $I^2 = 27.3\%$								

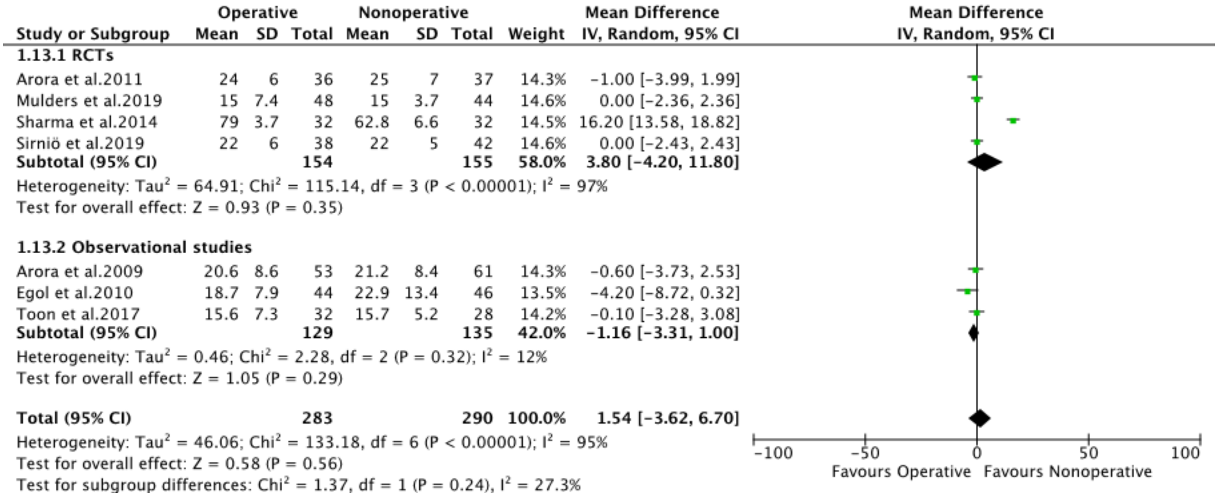

eFigure 16. Forest Plot of Ulnar Deviation in Degrees in a Meta-analysis of Distal Radius Fractures

Study or Subgroup	Operative Mean	Operative SD	Operative Total	Nonoperative Mean	Nonoperative SD	Nonoperative Total	Weight	Mean Difference IV, Random, 95% CI
**1.14.1 RCTs**								
Arora et al.2011	35	8	36	35	8	37	13.9%	0.00 [−3.67, 3.67]
Mulders et al.2019	25	4.4	48	25	7.4	44	14.6%	0.00 [−2.52, 2.52]
Sharma et al.2014	79.6	3	32	65.9	5.4	32	14.8%	13.70 [11.56, 15.84]
Sirniö et al.2019	28	5	38	25	6	42	14.6%	3.00 [0.59, 5.41]
**Subtotal (95% CI)**			**154**			**155**	**57.9%**	**4.23 [−2.76, 11.22]**
Heterogeneity: $Tau^2 = 48.92$; $Chi^2 = 87.45$, df = 3 (P < 0.00001); $I^2 = 97\%$								
Test for overall effect: Z = 1.19 (P = 0.24)								
**1.14.2 Observational studies**								
Arora et al.2009	38	9.4	53	36.4	9.2	61	14.0%	1.60 [−1.83, 5.03]
Egol et al.2010	29.9	8.8	44	30.3	7.1	46	14.1%	−0.40 [−3.71, 2.91]
Toon et al.2017	22.8	8	32	17.9	6	28	14.0%	4.90 [1.35, 8.45]
**Subtotal (95% CI)**			**129**			**135**	**42.1%**	**1.98 [−1.03, 4.99]**
Heterogeneity: $Tau^2 = 4.01$; $Chi^2 = 4.62$, df = 2 (P = 0.10); $I^2 = 57\%$								
Test for overall effect: Z = 1.29 (P = 0.20)								
**Total (95% CI)**			**283**			**290**	**100.0%**	**3.32 [−1.16, 7.79]**
Heterogeneity: $Tau^2 = 34.12$; $Chi^2 = 101.72$, df = 6 (P < 0.00001); $I^2 = 94\%$								
Test for overall effect: Z = 1.45 (P = 0.15)								
Test for subgroup differences: $Chi^2 = 0.34$, df = 1 (P = 0.56), $I^2 = 0\%$								

Mean Difference IV, Random, 95% CI

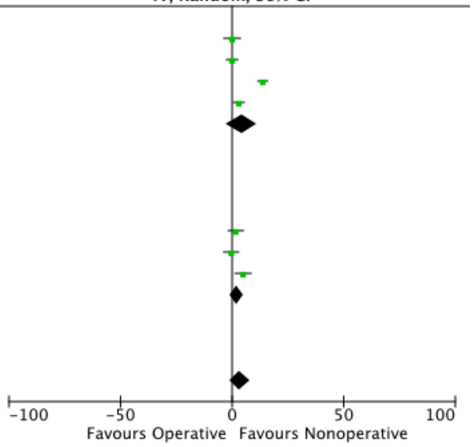

©2020 Ochen Y et al. *JAMA Network Open*.

eFigure 17. Forest Plot of Volar Tilt in Degrees in a Meta-analysis of Distal Radius Fractures

Study or Subgroup	Operative Mean	Operative SD	Operative Total	Nonoperative Mean	Nonoperative SD	Nonoperative Total	Weight	Mean Difference IV, Random, 95% CI
**1.15.1 RCTs**								
Arora et al.2011	3	7.2	36	−10.4	19.1	37	7.4%	13.40 [6.81, 19.99]
Martinez–Mendez et al.2018	8	8	50	4	8	47	12.0%	4.00 [0.81, 7.19]
Sharma et al.2014	8.3	1	32	5.1	0.5	32	14.7%	3.20 [2.81, 3.59]
**Subtotal (95% CI)**			**118**			**116**	**34.1%**	**5.44 [1.66, 9.22]**
Heterogeneity: $Tau^2 = 8.04$; $Chi^2 = 9.39$, df = 2 (P = 0.009); $I^2 = 79\%$								
Test for overall effect: Z = 2.82 (P = 0.005)								
**1.15.2 Observational studies**								
Egol et al.2010	6.2	9.2	44	−5.8	10.4	46	10.7%	12.00 [7.95, 16.05]
Gong et al.2011	8.1	1.5	26	5.3	3.4	24	14.1%	2.80 [1.32, 4.28]
Hung et al.2015	9.6	7.3	26	−5.2	15	31	8.1%	14.80 [8.82, 20.78]
Larouche et al.2016	8.3	7.9	70	1.3	12.3	59	11.3%	7.00 [3.36, 10.64]
Lutz et al.2014	2	9	129	7	13	129	12.6%	−5.00 [−7.73, −2.27]
Toon et al.2017	5.6	8.9	32	0.1	11.6	28	9.0%	5.50 [0.21, 10.79]
**Subtotal (95% CI)**			**327**			**317**	**65.9%**	**5.86 [0.80, 10.93]**
Heterogeneity: $Tau^2 = 35.80$; $Chi^2 = 72.44$, df = 5 (P < 0.00001); $I^2 = 93\%$								
Test for overall effect: Z = 2.27 (P = 0.02)								
**Total (95% CI)**			**445**			**433**	**100.0%**	**5.49 [2.94, 8.03]**
Heterogeneity: $Tau^2 = 11.41$; $Chi^2 = 81.87$, df = 8 (P < 0.00001); $I^2 = 90\%$								
Test for overall effect: Z = 4.23 (P < 0.0001)								
Test for subgroup differences: $Chi^2 = 0.02$, df = 1 (P = 0.90), $I^2 = 0\%$								

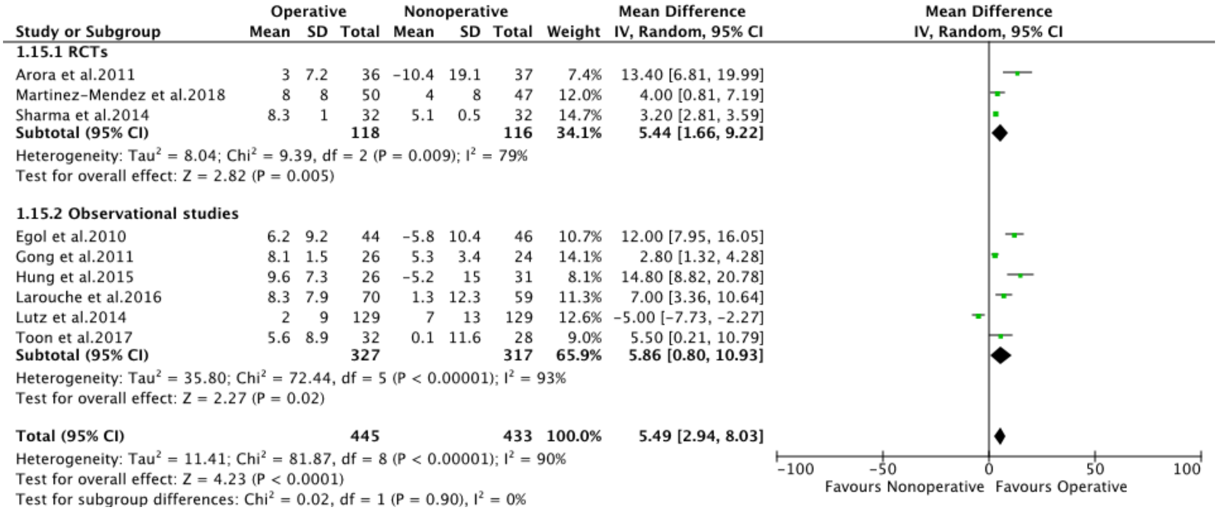

©2020 Ochen Y et al. *JAMA Network Open*.

eFigure 18. Forest Plot of Radial Inclination in Degrees in a Meta-analysis of Distal Radius Fractures

Study or Subgroup	Operative Mean	Operative SD	Operative Total	Nonoperative Mean	Nonoperative SD	Nonoperative Total	Weight	Mean Difference IV, Random, 95% CI
**1.16.1 RCTs**								
Arora et al.2011	21.2	2.6	36	15.9	9	37	4.2%	5.30 [2.28, 8.32]
Azzopardi et al.2005	22.5	5	27	19	6	27	4.4%	3.50 [0.55, 6.45]
Bartl et al.2014	20.3	4.5	68	17.7	6.3	81	8.2%	2.60 [0.86, 4.34]
Martinez-Mendez et al.2018	19	6	50	13	6	47	5.8%	6.00 [3.61, 8.39]
Sharma et al.2014	17.8	0.8	32	15.2	0.7	32	15.1%	2.60 [2.23, 2.97]
Sirniö et al.2019	24	4	38	21	8	42	4.9%	3.00 [0.27, 5.73]
**Subtotal (95% CI)**			**251**			**266**	**42.6%**	**3.48 [2.35, 4.62]**
Heterogeneity: $Tau^2 = 0.93$; $Chi^2 = 10.77$, df = 5 (P = 0.06); $I^2 = 54\%$								
Test for overall effect: Z = 6.03 (P < 0.00001)								
**1.16.2 Observational studies**								
Alm-Paulsen et al.2012	21	6	30	19	7	30	3.7%	2.00 [-1.30, 5.30]
Arora et al.2009	23.6	3.8	53	19.2	6.9	61	7.1%	4.40 [2.39, 6.41]
Egol et al.2010	22.3	4.7	44	18	4	46	7.9%	4.30 [2.49, 6.11]
Gong et al.2011	19.2	2.2	26	14.3	5.6	24	5.8%	4.90 [2.51, 7.29]
Hung et al.2015	21	4.6	26	17.9	5	31	5.5%	3.10 [0.61, 5.59]
Larouche et al.2016	24.2	5.2	70	21.2	6	59	7.3%	3.00 [1.04, 4.96]
Lutz et al.2014	20	5	129	19	6	129	10.2%	1.00 [-0.35, 2.35]
Toon et al.2017	21.6	6.1	32	16.9	6.3	28	4.0%	4.70 [1.55, 7.85]
Zengin et al.2019	21.5	2.6	25	16.6	5.3	24	5.9%	4.90 [2.55, 7.25]
**Subtotal (95% CI)**			**435**			**432**	**57.4%**	**3.50 [2.40, 4.60]**
Heterogeneity: $Tau^2 = 1.53$; $Chi^2 = 18.40$, df = 8 (P = 0.02); $I^2 = 57\%$								
Test for overall effect: Z = 6.22 (P < 0.00001)								
**Total (95% CI)**			**686**			**698**	**100.0%**	**3.46 [2.73, 4.18]**
Heterogeneity: $Tau^2 = 0.87$; $Chi^2 = 30.59$, df = 14 (P = 0.006); $I^2 = 54\%$								
Test for overall effect: Z = 9.35 (P < 0.00001)								
Test for subgroup differences: $Chi^2 = 0.00$, df = 1 (P = 0.99), $I^2 = 0\%$								

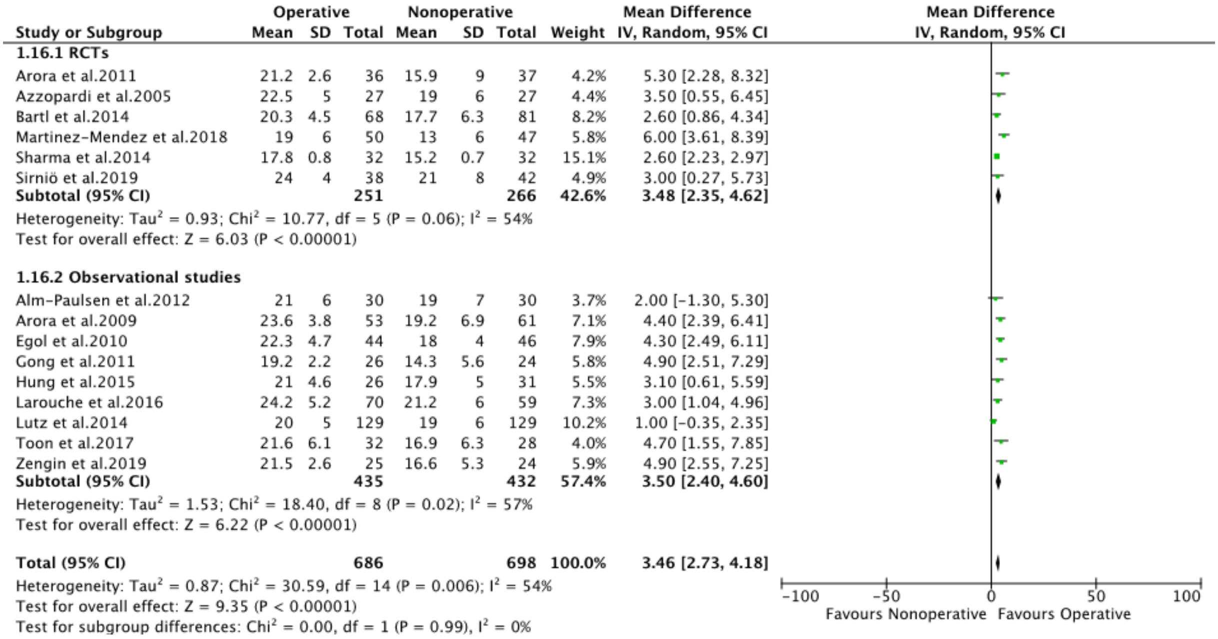

©2020 Ochen Y et al. *JAMA Network Open*.

eFigure 19. Forest Plot of Radial Height in Millimeters in a Meta-analysis of Distal Radius Fractures

Study or Subgroup	Operative Mean	Operative SD	Operative Total	Nonoperative Mean	Nonoperative SD	Nonoperative Total	Weight	Mean Difference IV, Random, 95% CI
**1.17.1 RCTs**								
Azzopardi et al.2005	8	3	27	5	4	27	5.0%	3.00 [1.11, 4.89]
Martinez–Mendez et al.2018	9	2	50	5	3	47	10.6%	4.00 [2.98, 5.02]
Sharma et al.2014	8.2	0.6	32	6.1	1.1	32	16.9%	2.10 [1.67, 2.53]
**Subtotal (95% CI)**			**109**			**106**	**32.6%**	**2.98 [1.58, 4.38]**
Heterogeneity: $Tau^2 = 1.20$; $Chi^2 = 11.65$, df = 2 (P = 0.003); $I^2 = 83\%$								
Test for overall effect: Z = 4.16 (P < 0.0001)								
**1.17.2 Observational studies**								
Alm–Paulsen et al.2012	7	4	30	6	5	30	3.7%	1.00 [−1.29, 3.29]
Egol et al.2010	10.6	2.5	44	8.7	1.6	46	12.1%	1.90 [1.03, 2.77]
Gong et al.2011	9.5	1.3	26	6.3	3.2	24	7.7%	3.20 [1.83, 4.57]
Hung et al.2015	9.8	2.4	26	8.6	2.6	31	8.3%	1.20 [−0.10, 2.50]
Jordan et al.2016	10.9	2.6	74	8.2	2.2	85	13.4%	2.70 [1.95, 3.45]
Larouche et al.2016	11.1	3.4	70	9.7	3.4	59	9.2%	1.40 [0.22, 2.58]
Toon et al.2017	9.6	3.3	32	7.2	3.4	28	5.9%	2.40 [0.70, 4.10]
Zengin et al.2019	10.4	2.8	25	7.8	2.4	24	7.2%	2.60 [1.14, 4.06]
**Subtotal (95% CI)**			**327**			**327**	**67.4%**	**2.14 [1.63, 2.65]**
Heterogeneity: $Tau^2 = 0.14$; $Chi^2 = 9.61$, df = 7 (P = 0.21); $I^2 = 27\%$								
Test for overall effect: Z = 8.28 (P < 0.00001)								
**Total (95% CI)**			**436**			**433**	**100.0%**	**2.36 [1.87, 2.85]**
Heterogeneity: $Tau^2 = 0.32$; $Chi^2 = 21.93$, df = 10 (P = 0.02); $I^2 = 54\%$								
Test for overall effect: Z = 9.41 (P < 0.00001)								
Test for subgroup differences: $Chi^2 = 1.21$, df = 1 (P = 0.27), $I^2 = 17.6\%$								

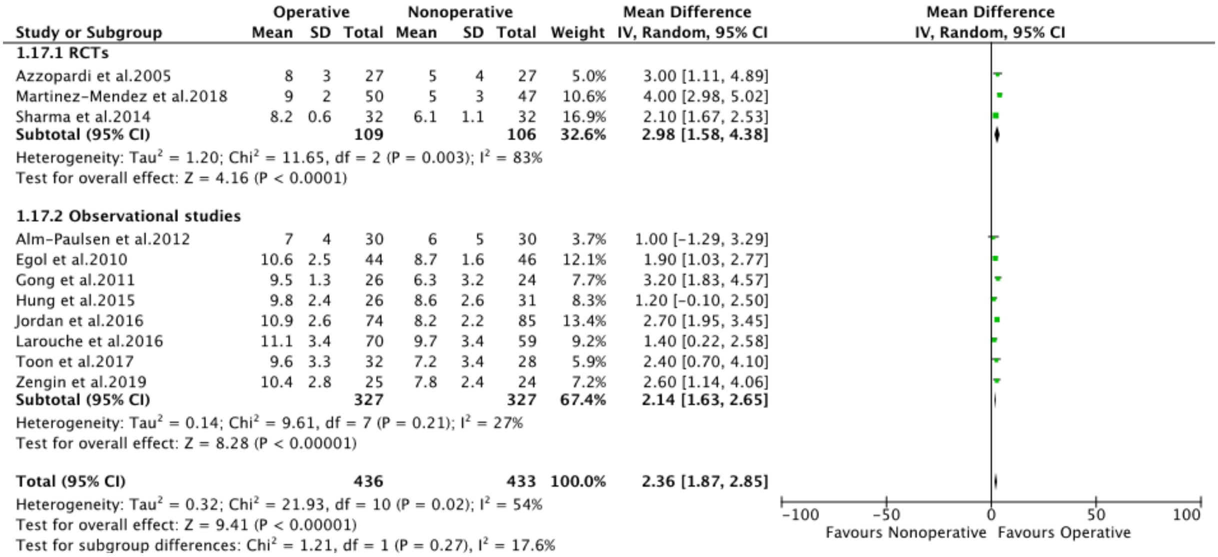

©2020 Ochen Y et al. *JAMA Network Open*.

eFigure 20. Forest Plot Of Articular Step-off in Millimeters in a Meta-analysis of Distal Radius Fractures

Study or Subgroup	Operative Mean	Operative SD	Operative Total	Nonoperative Mean	Nonoperative SD	Nonoperative Total	Weight	Mean Difference IV, Random, 95% CI
**1.18.1 RCTs**								
Arora et al.2011	0.2	0.5	36	0.6	1.1	37	13.1%	−0.40 [−0.79, −0.01]
Martinez–Mendez et al.2018	0.4	1	50	1	3	47	5.1%	−0.60 [−1.50, 0.30]
**Subtotal (95% CI)**			**86**			**84**	**18.2%**	**−0.43 [−0.79, −0.07]**
Heterogeneity: $Tau^2 = 0.00$; $Chi^2 = 0.16$, df = 1 (P = 0.69); $I^2 = 0\%$								
Test for overall effect: Z = 2.36 (P = 0.02)								
**1.18.2 Observational studies**								
Jordan et al.2016	0.9	0.5	74	0.9	0.6	85	18.4%	0.00 [−0.17, 0.17]
Larouche et al.2016	0.1	0.4	70	0.1	0.4	59	19.0%	0.00 [−0.14, 0.14]
Lutz et al.2014	0.3	0.7	129	0.2	0.6	129	18.7%	0.10 [−0.06, 0.26]
Toon et al.2017	0.7	0.6	32	1.5	0.9	28	13.0%	−0.80 [−1.19, −0.41]
Zengin et al.2019	0.6	0.5	25	1.4	0.9	24	12.6%	−0.80 [−1.21, −0.39]
**Subtotal (95% CI)**			**330**			**325**	**81.8%**	**−0.23 [−0.49, 0.03]**
Heterogeneity: $Tau^2 = 0.07$; $Chi^2 = 31.08$, df = 4 (P < 0.00001); $I^2 = 87\%$								
Test for overall effect: Z = 1.71 (P = 0.09)								
**Total (95% CI)**			**416**			**409**	**100.0%**	**−0.27 [−0.51, −0.03]**
Heterogeneity: $Tau^2 = 0.07$; $Chi^2 = 35.51$, df = 6 (P < 0.00001); $I^2 = 83\%$								
Test for overall effect: Z = 2.24 (P = 0.03)								
Test for subgroup differences: $Chi^2 = 0.80$, df = 1 (P = 0.37), $I^2 = 0\%$								

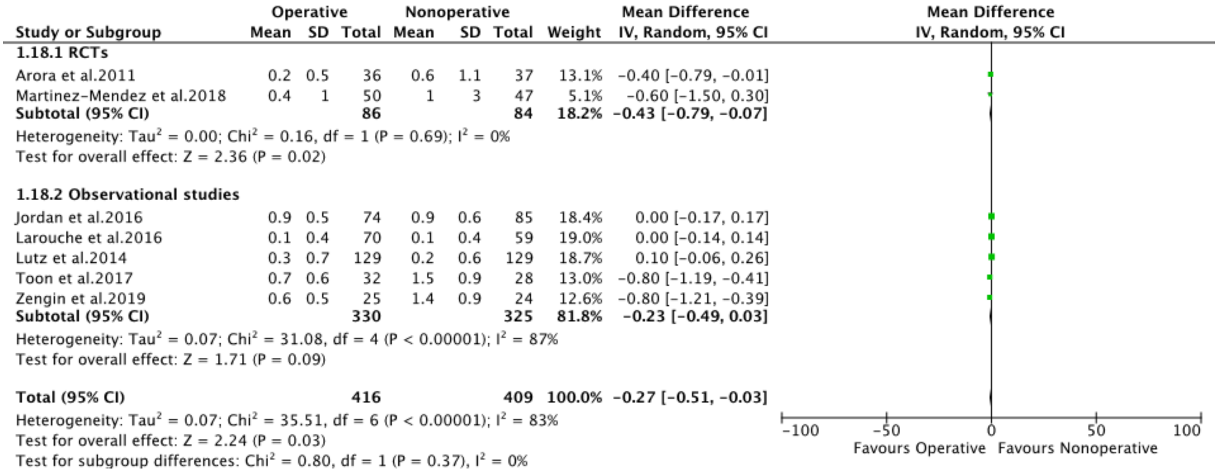

eFigure 21. Forest Plot of Ulnar Variance in Millimeters in a Meta-analysis of Distal Radius Fractures

	Operative			Nonoperative				Mean Difference
**Study or Subgroup**	**Mean**	**SD**	**Total**	**Mean**	**SD**	**Total**	**Weight**	**IV, Random, 95% CI**
**1.19.1 RCTs**								
Arora et al.2011	0.7	1.8	36	3.2	2.9	37	8.4%	−2.50 [−3.60, −1.40]
Azzopardi et al.2005	3	2	27	3	2	27	8.5%	0.00 [−1.07, 1.07]
Martinez-Mendez et al.2018	4	3	50	1	2	47	8.7%	3.00 [1.99, 4.01]
Sharma et al.2014	−0.2	0.2	32	0.2	0.1	32	10.7%	−0.40 [−0.48, −0.32]
Sirniö et al.2019	−0.7	0.9	38	−2	1.7	42	9.9%	1.30 [0.71, 1.89]
**Subtotal (95% CI)**			**183**			**185**	**46.2%**	**0.29 [−1.01, 1.60]**
Heterogeneity: $Tau^2 = 2.03$; $Chi^2 = 89.05$, df = 4 (P < 0.00001); $I^2 = 96\%$								
Test for overall effect: Z = 0.44 (P = 0.66)								
**1.19.2 Observational studies**								
Alm-Paulsen et al.2012	3	2	30	3	3	30	7.8%	0.00 [−1.29, 1.29]
Arora et al.2009	1.5	1.9	53	3.8	2.6	61	9.3%	−2.30 [−3.13, −1.47]
Egol et al.2010	1.5	2.2	44	2.8	1.8	46	9.2%	−1.30 [−2.13, −0.47]
Larouche et al.2016	−0.8	2.4	70	−1.6	2.8	59	9.0%	0.80 [−0.11, 1.71]
Lutz et al.2014	1.3	2.1	129	2.6	2.2	129	10.1%	−1.30 [−1.82, −0.78]
Zengin et al.2019	1.7	1.7	25	2.1	2.1	24	8.5%	−0.40 [−1.47, 0.67]
**Subtotal (95% CI)**			**351**			**349**	**53.8%**	**−0.80 [−1.65, 0.05]**
Heterogeneity: $Tau^2 = 0.92$; $Chi^2 = 29.76$, df = 5 (P < 0.0001); $I^2 = 83\%$								
Test for overall effect: Z = 1.84 (P = 0.07)								
**Total (95% CI)**			**534**			**534**	**100.0%**	**−0.29 [−0.97, 0.40]**
Heterogeneity: $Tau^2 = 1.14$; $Chi^2 = 133.10$, df = 10 (P < 0.00001); $I^2 = 92\%$								
Test for overall effect: Z = 0.83 (P = 0.41)								
Test for subgroup differences: $Chi^2 = 1.88$, df = 1 (P = 0.17), $I^2 = 46.8\%$								

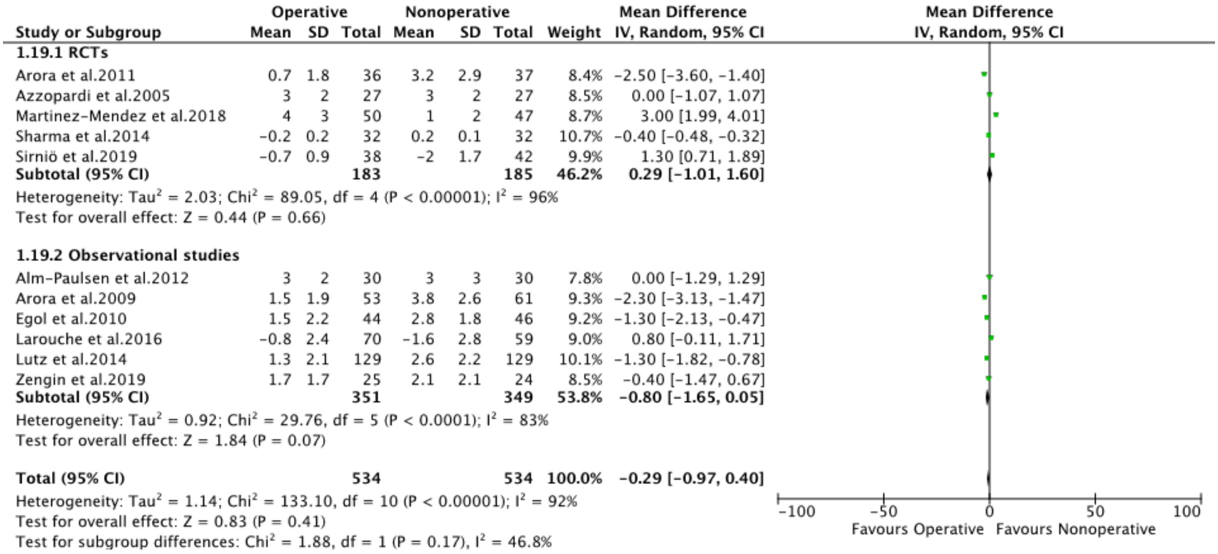

©2020 Ochen Y et al. *JAMA Network Open*.

eFigure 22. Forest Plot of Medium-Term (≤1 y) DASH Score for Studies That Only Included Patients With Age >60 Years and Other Studies That Included Patients With Age ≥18 Years in a Meta-analysis of Distal Radius Fractures

	Operative			Nonoperative				Mean Difference
Study or Subgroup	Mean	SD	Total	Mean	SD	Total	Weight	IV, Random, 95% CI
**1.29.1 Studies only age >60 years**								
Arora et al.2011	5.7	11.1	36	8	9.3	37	11.2%	−2.30 [−7.00, 2.40]
Bartl et al.2014	14	16.1	68	19	21.3	81	10.0%	−5.00 [−11.01, 1.01]
Chan et al.2014	6.7	1.9	40	6.2	1.9	35	13.6%	0.50 [−0.36, 1.36]
Egol et al.2010	10	20.3	44	12.1	29.6	46	6.5%	−2.10 [−12.55, 8.35]
**Subtotal (95% CI)**			**188**			**199**	**41.2%**	**−0.98 [−3.52, 1.57]**
Heterogeneity: $Tau^2 = 2.59$; $Chi^2 = 4.57$, df = 3 (P = 0.21); $I^2 = 34\%$								
Test for overall effect: Z = 0.75 (P = 0.45)								
**1.29.2 Other studies age >18 years**								
Gong et al.2011	13.9	8.8	26	25.9	14	24	9.5%	−12.00 [−18.54, −5.46]
Larouche et al.2016	10.9	14.7	70	11	11.9	59	11.3%	−0.10 [−4.69, 4.49]
Mulders et al.2019	2.5	9.4	48	9.2	10.8	44	11.6%	−6.70 [−10.85, −2.55]
Sharma et al.2014	4.9	9.4	32	14	10.2	32	11.1%	−9.10 [−13.91, −4.29]
Tan et al.2012	9	12	31	28	22	32	7.7%	−19.00 [−27.71, −10.29]
Toon et al.2017	16.2	17.4	32	16.1	17.7	28	7.6%	0.10 [−8.81, 9.01]
**Subtotal (95% CI)**			**239**			**219**	**58.8%**	**−7.50 [−12.40, −2.60]**
Heterogeneity: $Tau^2 = 27.29$; $Chi^2 = 21.31$, df = 5 (P = 0.0007); $I^2 = 77\%$								
Test for overall effect: Z = 3.00 (P = 0.003)								
**Total (95% CI)**			**427**			**418**	**100.0%**	**−5.22 [−8.87, −1.57]**
Heterogeneity: $Tau^2 = 25.30$; $Chi^2 = 58.04$, df = 9 (P < 0.00001); $I^2 = 84\%$								
Test for overall effect: Z = 2.80 (P = 0.005)								
Test for subgroup differences: $Chi^2 = 5.37$, df = 1 (P = 0.02), $I^2 = 81.4\%$								

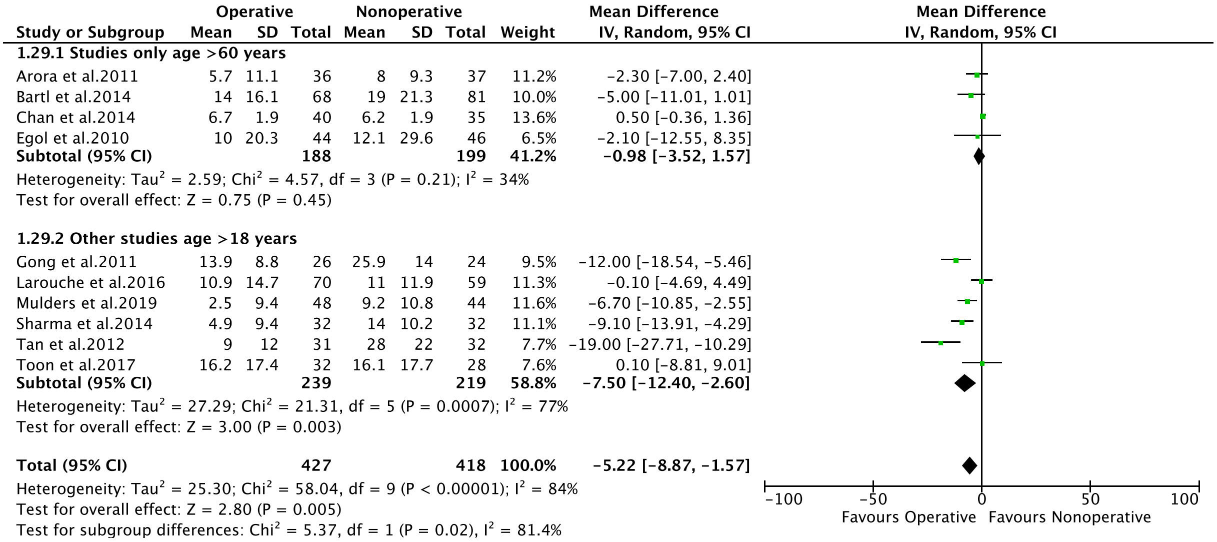

©2020 Ochen Y et al. *JAMA Network Open*.

eFigure 23. Forest Plot of Complication Rate for Studies That Only Included Patients With Age >60 Years and Other Studies That Included Patients With Age ≥18 Years in a Meta-analysis of Distal Radius Fractures

Study or Subgroup	Operative Events	Operative Total	Nonoperative Events	Nonoperative Total	Weight	Risk Ratio M-H, Random, 95% CI
**1.30.1 Studies only age >60 years**						
Aktekin et al.2010	7	22	10	24	8.0%	0.76 [0.35, 1.66]
Arora et al.2009	7	53	5	61	6.2%	1.61 [0.54, 4.78]
Arora et al.2011	13	36	5	37	7.1%	2.67 [1.06, 6.73]
Azzopardi et al.2005	1	27	1	27	1.9%	1.00 [0.07, 15.18]
Bartl et al.2014	8	68	10	81	7.4%	0.95 [0.40, 2.28]
Chan et al.2014	5	40	4	35	5.5%	1.09 [0.32, 3.76]
Egol et al.2010	7	44	4	46	5.9%	1.83 [0.58, 5.82]
Hung et al.2015	0	26	1	31	1.4%	0.40 [0.02, 9.31]
Lutz et al.2014	50	129	27	129	10.1%	1.85 [1.24, 2.76]
Martinez–Mendez et al.2018	2	50	1	47	2.3%	1.88 [0.18, 20.05]
**Subtotal (95% CI)**		**495**		**518**	**55.8%**	**1.51 [1.15, 2.00]**
Total events	100		68			
Heterogeneity: Tau² = 0.00; Chi² = 7.72, df = 9 (P = 0.56); I² = 0%						
Test for overall effect: Z = 2.94 (P = 0.003)						
**1.30.2 Other studies age >18 years**						
Abbaszadegan et al.1990	4	23	0	24	1.7%	9.38 [0.53, 164.94]
Alm–Paulsen et al.2012	6	30	3	30	5.3%	2.00 [0.55, 7.27]
Barai et al.2018	1	29	7	87	2.9%	0.43 [0.06, 3.34]
Gong et al.2011	1	26	0	24	1.4%	2.78 [0.12, 65.08]
Mulders et al.2019	16	48	15	44	9.2%	0.98 [0.55, 1.73]
Sharma et al.2014	8	32	29	32	8.9%	0.28 [0.15, 0.51]
Sirniö et al.2019	3	38	5	42	5.0%	0.66 [0.17, 2.59]
Tan et al.2012	7	31	20	32	8.4%	0.36 [0.18, 0.73]
Toon et al.2017	1	32	0	28	1.4%	2.64 [0.11, 62.23]
**Subtotal (95% CI)**		**289**		**343**	**44.2%**	**0.73 [0.39, 1.38]**
Total events	47		79			
Heterogeneity: Tau² = 0.43; Chi² = 19.93, df = 8 (P = 0.01); I² = 60%						
Test for overall effect: Z = 0.96 (P = 0.34)						
**Total (95% CI)**		**784**		**861**	**100.0%**	**1.03 [0.69, 1.55]**
Total events	147		147			
Heterogeneity: Tau² = 0.38; Chi² = 46.78, df = 18 (P = 0.0002); I² = 62%						
Test for overall effect: Z = 0.17 (P = 0.87)						
Test for subgroup differences: Chi² = 4.22, df = 1 (P = 0.04), I² = 76.3%						

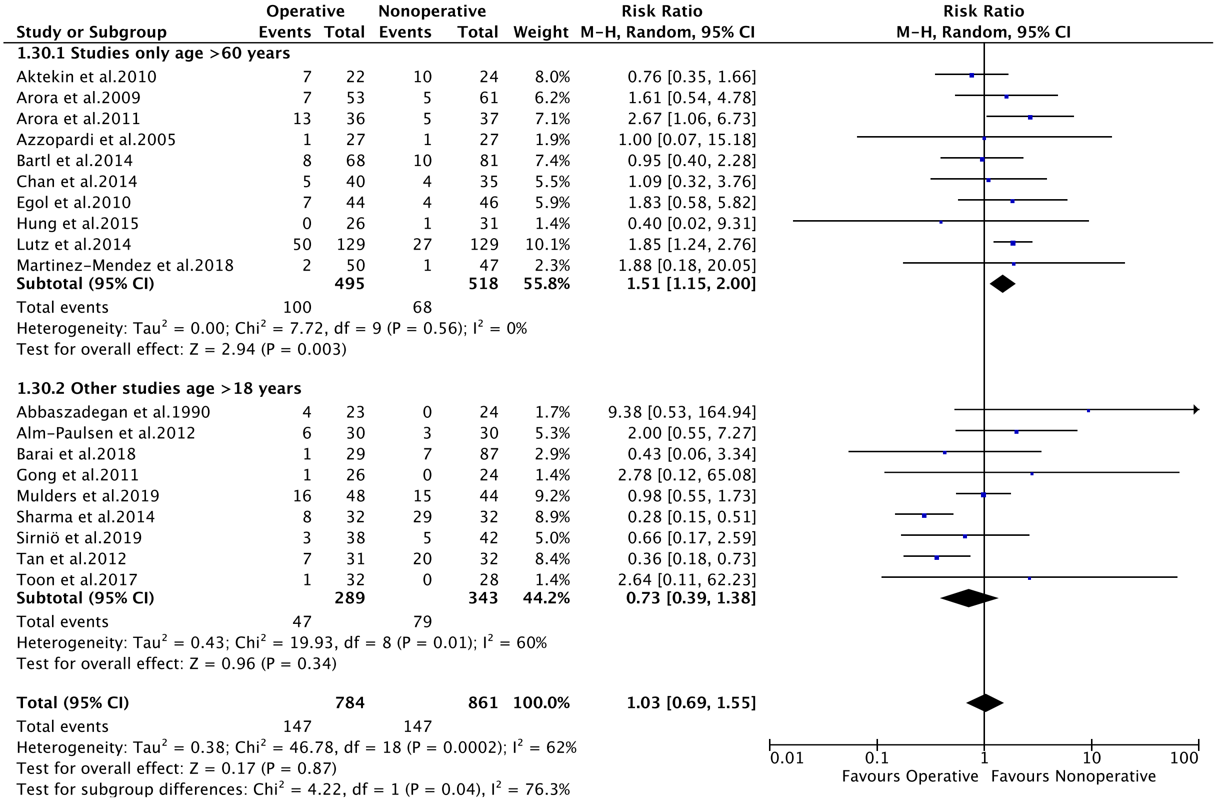

©2020 Ochen Y et al. *JAMA Network Open*.

eFigure 24. Forest Plot of Medium-Term (≤1 y) DASH Score in High-Quality Studies in a Meta-analysis of Distal Radius Fractures

	Operative			Nonoperative				Mean Difference
**Study or Subgroup**	**Mean**	**SD**	**Total**	**Mean**	**SD**	**Total**	**Weight**	**IV, Random, 95% CI**
**1.23.1 RCTs**								
Arora et al.2011	5.7	11.1	36	8	9.3	37	14.9%	−2.30 [−7.00, 2.40]
Bartl et al.2014	14	16.1	68	19	21.3	81	13.6%	−5.00 [−11.01, 1.01]
Mulders et al.2019	2.5	9.4	48	9.2	10.8	44	15.3%	−6.70 [−10.85, −2.55]
Sharma et al.2014	4.9	9.4	32	14	10.2	32	14.8%	−9.10 [−13.91, −4.29]
**Subtotal (95% CI)**			**184**			**194**	**58.6%**	**−5.86 [−8.71, −3.00]**
Heterogeneity: $Tau^2 = 2.40$; $Chi^2 = 4.18$, df = 3 (P = 0.24); $I^2 = 28\%$								
Test for overall effect: Z = 4.02 (P < 0.0001)								
**1.23.2 Observational studies**								
Chan et al.2014	6.7	1.9	40	6.2	1.9	35	17.2%	0.50 [−0.36, 1.36]
Gong et al.2011	13.9	8.8	26	25.9	14	24	13.1%	−12.00 [−18.54, −5.46]
Tan et al.2012	9	12	31	28	22	32	11.1%	−19.00 [−27.71, −10.29]
**Subtotal (95% CI)**			**97**			**91**	**41.4%**	**−9.63 [−22.05, 2.79]**
Heterogeneity: $Tau^2 = 110.64$; $Chi^2 = 32.42$, df = 2 (P < 0.00001); $I^2 = 94\%$								
Test for overall effect: Z = 1.52 (P = 0.13)								
**Total (95% CI)**			**281**			**285**	**100.0%**	**−6.98 [−11.80, −2.17]**
Heterogeneity: $Tau^2 = 34.90$; $Chi^2 = 57.89$, df = 6 (P < 0.00001); $I^2 = 90\%$								
Test for overall effect: Z = 2.84 (P = 0.004)								
Test for subgroup differences: $Chi^2 = 0.34$, df = 1 (P = 0.56), $I^2 = 0\%$								

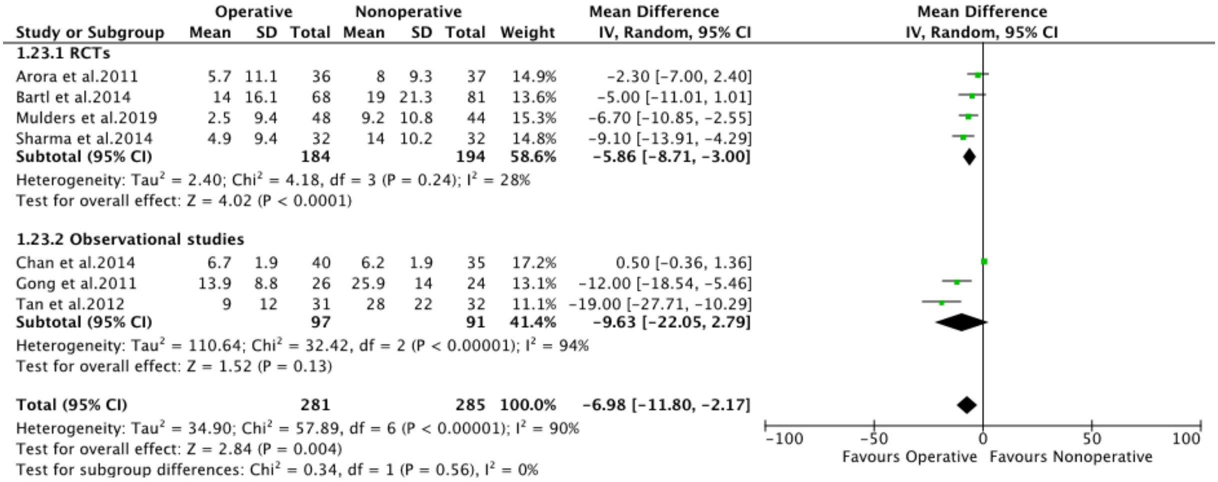

©2020 Ochen Y et al. *JAMA Network Open*.

eFigure 25. Forest Plot of Complication Rate in High-Quality Studies in a Meta-analysis of Distal Radius Fractures

Study or Subgroup	Operative Events	Operative Total	Nonoperative Events	Nonoperative Total	Weight	Risk Ratio M-H, Random, 95% CI
**1.27.1 RCTs**						
Abbaszadegan et al.1990	4	23	0	24	3.2%	9.38 [0.53, 164.94]
Arora et al.2011	13	36	5	37	12.0%	2.67 [1.06, 6.73]
Azzopardi et al.2005	1	27	1	27	3.4%	1.00 [0.07, 15.18]
Bartl et al.2014	8	68	10	81	12.5%	0.95 [0.40, 2.28]
Martinez-Mendez et al.2018	2	50	1	47	4.3%	1.88 [0.18, 20.05]
Mulders et al.2019	16	48	15	44	15.0%	0.98 [0.55, 1.73]
Sharma et al.2014	8	32	29	32	14.7%	0.28 [0.15, 0.51]
Sirniö et al.2019	3	38	5	42	8.7%	0.66 [0.17, 2.59]
**Subtotal (95% CI)**		**322**		**334**	**73.8%**	**1.00 [0.49, 2.01]**
Total events	55		66			
Heterogeneity: $Tau^2 = 0.57$; $Chi^2 = 22.50$, df = 7 (P = 0.002); $I^2 = 69\%$						
Test for overall effect: Z = 0.01 (P = 0.99)						
**1.27.2 Observational studies**						
Chan et al.2014	5	40	4	35	9.6%	1.09 [0.32, 3.76]
Gong et al.2011	1	26	0	24	2.7%	2.78 [0.12, 65.08]
Tan et al.2012	7	31	20	32	13.9%	0.36 [0.18, 0.73]
**Subtotal (95% CI)**		**97**		**91**	**26.2%**	**0.64 [0.23, 1.77]**
Total events	13		24			
Heterogeneity: $Tau^2 = 0.35$; $Chi^2 = 3.54$, df = 2 (P = 0.17); $I^2 = 44\%$						
Test for overall effect: Z = 0.85 (P = 0.39)						
**Total (95% CI)**		**419**		**425**	**100.0%**	**0.88 [0.50, 1.55]**
Total events	68		90			
Heterogeneity: $Tau^2 = 0.46$; $Chi^2 = 27.59$, df = 10 (P = 0.002); $I^2 = 64\%$						
Test for overall effect: Z = 0.43 (P = 0.66)						
Test for subgroup differences: $Chi^2 = 0.48$, df = 1 (P = 0.49), $I^2 = 0\%$						

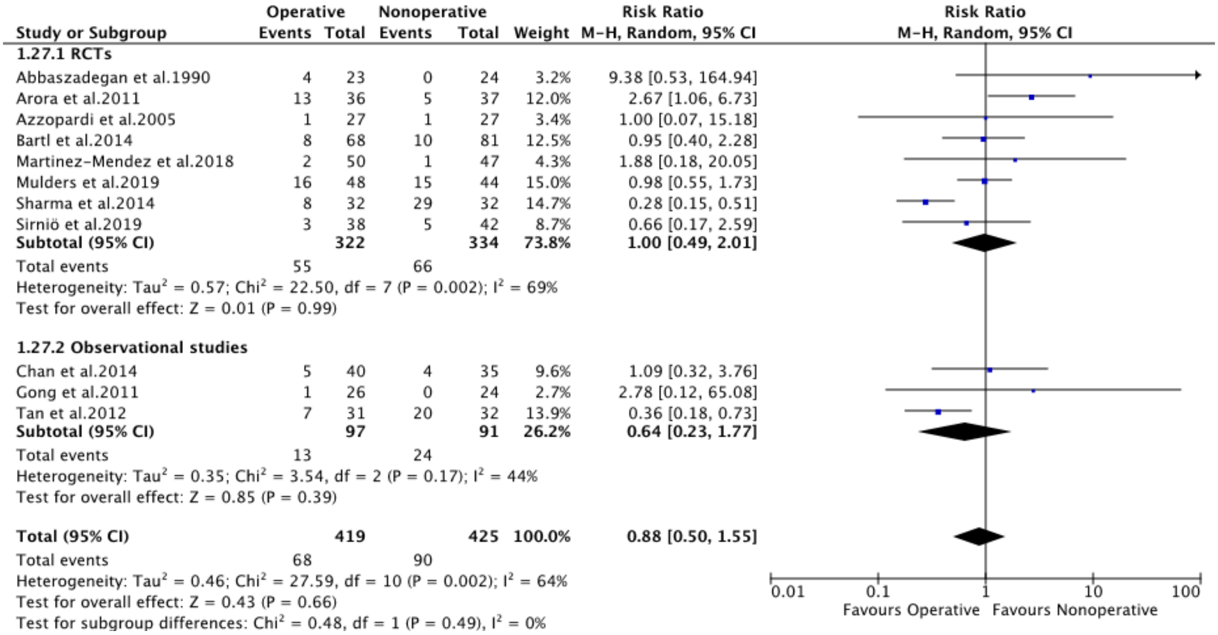

eFigure 26. Forest Plot of Medium-Term (≤1 y) DASH Score in Studies With a Study Period After 2008 in a Meta-analysis of Distal Radius Fractures

Study or Subgroup	Operative Mean	Operative SD	Operative Total	Nonoperative Mean	Nonoperative SD	Nonoperative Total	Weight	Mean Difference IV, Random, 95% CI
**1.24.1 RCTs**								
Bartl et al.2014	14	16.1	68	19	21.3	81	15.9%	−5.00 [−11.01, 1.01]
Mulders et al.2019	2.5	9.4	48	9.2	10.8	44	18.2%	−6.70 [−10.85, −2.55]
Sharma et al.2014	4.9	9.4	32	14	10.2	32	17.4%	−9.10 [−13.91, −4.29]
**Subtotal (95% CI)**			**148**			**157**	**51.6%**	**−7.14 [−9.93, −4.36]**
Heterogeneity: Tau² = 0.00; Chi² = 1.17, df = 2 (P = 0.56); I² = 0%								
Test for overall effect: Z = 5.03 (P < 0.00001)								
**1.24.2 Observational studies**								
Chan et al.2014	6.7	1.9	40	6.2	1.9	35	20.8%	0.50 [−0.36, 1.36]
Gong et al.2011	13.9	8.8	26	25.9	14	24	15.2%	−12.00 [−18.54, −5.46]
Toon et al.2017	16.2	17.4	32	16.1	17.7	28	12.4%	0.10 [−8.81, 9.01]
**Subtotal (95% CI)**			**98**			**87**	**48.4%**	**−3.64 [−11.87, 4.59]**
Heterogeneity: Tau² = 43.52; Chi² = 13.78, df = 2 (P = 0.001); I² = 85%								
Test for overall effect: Z = 0.87 (P = 0.39)								
**Total (95% CI)**			**246**			**244**	**100.0%**	**−5.31 [−10.20, −0.43]**
Heterogeneity: Tau² = 29.59; Chi² = 39.93, df = 5 (P < 0.00001); I² = 87%								
Test for overall effect: Z = 2.13 (P = 0.03)								
Test for subgroup differences: Chi² = 0.62, df = 1 (P = 0.43), I² = 0%								

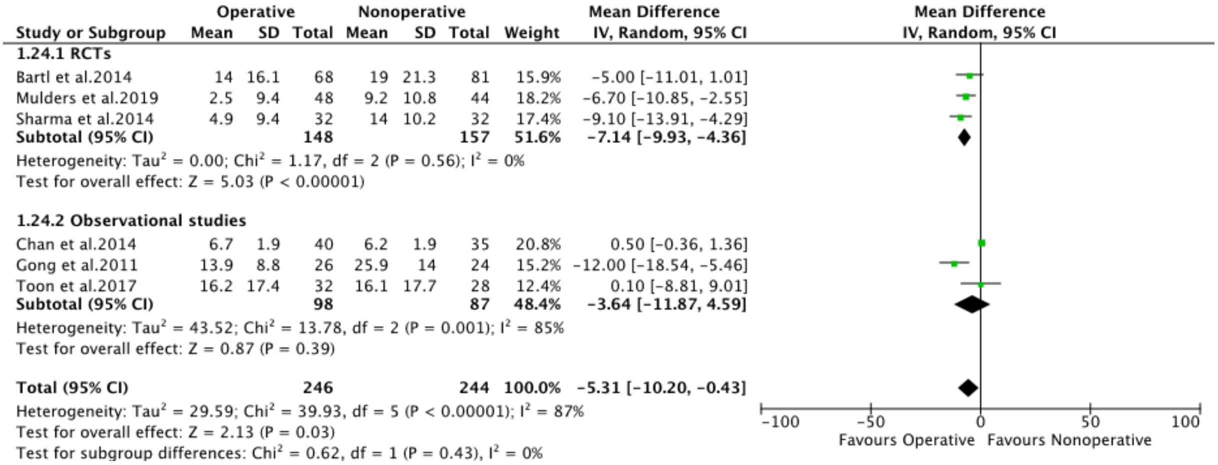

©2020 Ochen Y et al. *JAMA Network Open*.

eFigure 27. Forest Plot of Complication Rate in Studies With a Study Period After 2008 in a Meta-analysis of Distal Radius Fractures

	Operative		Nonoperative			Risk Ratio
**Study or Subgroup**	**Events**	**Total**	**Events**	**Total**	**Weight**	**M–H, Random, 95% CI**
**1.28.1 RCTs**						
Bartl et al.2014	8	68	10	81	16.8%	0.95 [0.40, 2.28]
Martinez–Mendez et al.2018	2	50	1	47	3.8%	1.88 [0.18, 20.05]
Mulders et al.2019	16	48	15	44	24.1%	0.98 [0.55, 1.73]
Sharma et al.2014	8	32	29	32	23.1%	0.28 [0.15, 0.51]
Sirniö et al.2019	3	38	5	42	9.5%	0.66 [0.17, 2.59]
**Subtotal (95% CI)**		**236**		**246**	**77.4%**	**0.68 [0.34, 1.33]**
Total events	37		60			
Heterogeneity: $Tau^2 = 0.34$; $Chi^2 = 10.97$, df = 4 (P = 0.03); $I^2 = 64\%$						
Test for overall effect: Z = 1.13 (P = 0.26)						
**1.28.2 Observational studies**						
Barai et al.2018	1	29	7	87	4.9%	0.43 [0.06, 3.34]
Chan et al.2014	5	40	4	35	11.0%	1.09 [0.32, 3.76]
Gong et al.2011	1	26	0	24	2.3%	2.78 [0.12, 65.08]
Hung et al.2015	0	26	1	31	2.3%	0.40 [0.02, 9.31]
Toon et al.2017	1	32	0	28	2.2%	2.64 [0.11, 62.23]
**Subtotal (95% CI)**		**153**		**205**	**22.6%**	**0.97 [0.39, 2.42]**
Total events	8		12			
Heterogeneity: $Tau^2 = 0.00$; $Chi^2 = 1.77$, df = 4 (P = 0.78); $I^2 = 0\%$						
Test for overall effect: Z = 0.06 (P = 0.95)						
**Total (95% CI)**		**389**		**451**	**100.0%**	**0.72 [0.44, 1.17]**
Total events	45		72			
Heterogeneity: $Tau^2 = 0.17$; $Chi^2 = 13.54$, df = 9 (P = 0.14); $I^2 = 34\%$						
Test for overall effect: Z = 1.34 (P = 0.18)						
Test for subgroup differences: $Chi^2 = 0.39$, df = 1 (P = 0.53), $I^2 = 0\%$						

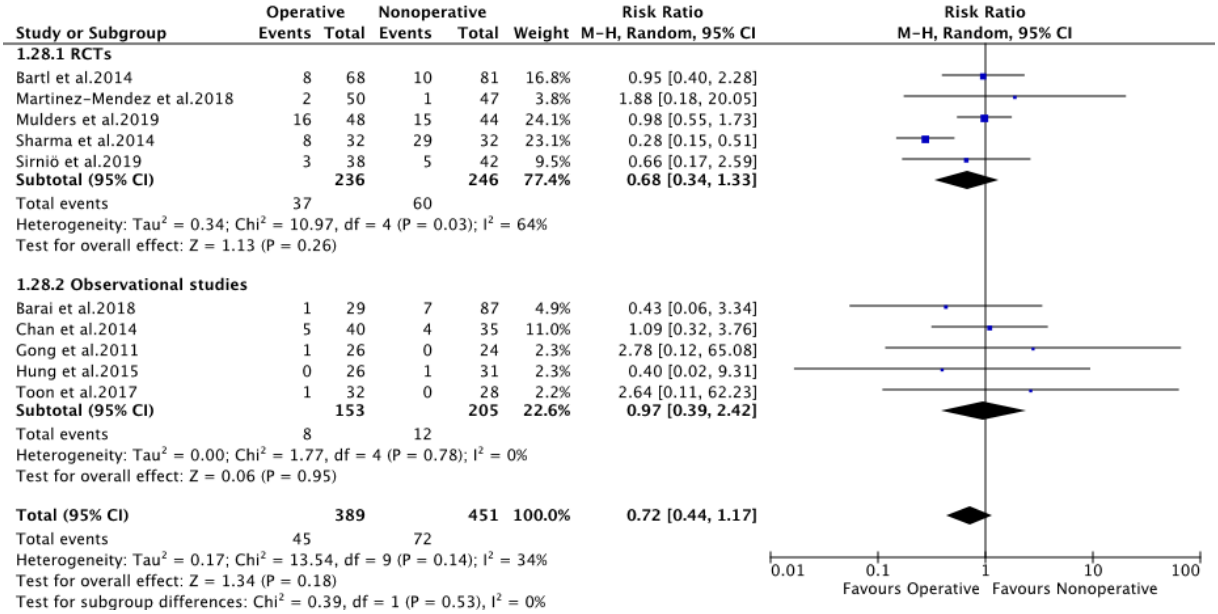

©2020 Ochen Y et al. *JAMA Network Open*.
